# SKP2- and OTUD1-regulated non-proteolytic ubiquitination of YAP promotes YAP nuclear localization and activity

**DOI:** 10.1038/s41467-018-04620-y

**Published:** 2018-06-11

**Authors:** Fan Yao, Zhicheng Zhou, Jongchan Kim, Qinglei Hang, Zhenna Xiao, Baochau N. Ton, Liang Chang, Na Liu, Liyong Zeng, Wenqi Wang, Yumeng Wang, Peijing Zhang, Xiaoyu Hu, Xiaohua Su, Han Liang, Yutong Sun, Li Ma

**Affiliations:** 1 Department of Experimental Radiation Oncology, The University of Texas MD Anderson Cancer Center, Houston, TX 77030 USA; 2 The University of Texas MD Anderson Cancer Center UTHealth Graduate School of Biomedical Sciences, Houston, TX 77030 USA; 3 Department of Developmental and Cell Biology, University of California, Irvine, CA 92697 USA; 4 Department of Bioinformatics and Computational Biology, The University of Texas MD Anderson Cancer Center, Houston, TX 77030 USA; 5 Key Laboratory of Molecular Biophysics of the Ministry of Education, College of Life Science and Technology, Huazhong University of Science and Technology, Wuhan, Hubei 430074 China; 6 Department of Molecular and Cellular Oncology, The University of Texas MD Anderson Cancer Center, Houston, TX 77030 USA

## Abstract

Dysregulation of YAP localization and activity is associated with pathological conditions such as cancer. Although activation of the Hippo phosphorylation cascade is known to cause cytoplasmic retention and inactivation of YAP, emerging evidence suggests that YAP can be regulated in a Hippo-independent manner. Here, we report that YAP is subject to non-proteolytic, K63-linked polyubiquitination by the SCF^SKP2^ E3 ligase complex (SKP2), which is reversed by the deubiquitinase OTUD1. The non-proteolytic ubiquitination of YAP enhances its interaction with its nuclear binding partner TEAD, thereby inducing YAP’s nuclear localization, transcriptional activity, and growth-promoting function. Independently of Hippo signaling, mutation of YAP’s K63-linkage specific ubiquitination sites K321 and K497, depletion of SKP2, or overexpression of OTUD1 retains YAP in the cytoplasm and inhibits its activity. Conversely, overexpression of SKP2 or loss of OTUD1 leads to nuclear localization and activation of YAP. Altogether, our study sheds light on the ubiquitination-mediated, Hippo-independent regulation of YAP.

## Abstract (web summary)

Regulation of Yes-associated protein (YAP) through the Hippo pathway is well established, but its Hippo-independent regulation remains to be elucidated. Here, the authors show that non-proteolytic ubiquitination presents another means of YAP regulation, promoting its nuclear localization and activity.

## Introduction

Yes-associated protein (YAP) is a key player in regulating organ size, tissue homeostasis, and tumorigenesis^1^. In mice, intestine-specific or heart-specific deletion of *Yap* impeded intestinal regeneration^2^ and neonatal cardiac regeneration^3^ after tissue injury, respectively, while transgenic overexpression of *YAP* led to enlarged liver that ultimately progressed to hepatocellular carcinoma^4,5^. Moreover, overexpression of YAP in melanoma and breast cancer cells promoted tumor growth and metastasis^6^, while genetic ablation of *Yap* in mouse cancer models inhibited liver and mammary tumorigenesis^7,8^.

YAP shuttles between the cytoplasm and the nucleus of the cell, and its subcellular localization determines its activity^9^. In the nucleus, YAP acts as a transcriptional co-activator that interacts with transcription factors, particularly TEA domain (TEAD) family members, to regulate the expression of genes important for cell proliferation, apoptosis, and migration, such as *CTGF*, *CYR61*, *ANKRD1*, *AREG*, and *BIRC5*^10–12^. Dysregulation of YAP is prevalent in various types of cancer in which YAP contributes to tumor initiation, progression and/or metastasis^13^. In addition to amplification and overexpression of *YAP*^14,15^, YAP protein has been found to accumulate in the nucleus of many human tumor cells^16^. Therefore, it is important to understand the regulation of YAP’s subcellular localization and activity under physiological and pathological conditions.

Post-translational modifications of YAP regulate its stability, activity, subcellular localization, and interaction with other proteins^9^. Conserved from *Drosophila* to mammals, the Hippo pathway regulates YAP localization and activity via phosphorylation^4^. In human cells, various upstream signals provide inputs that feed into the MST1/2 (mammalian Hippo homologs) substrates, LATS1/2, to phosphorylate YAP at serine 127 (Ser127), leading to its binding to 14-3-3, which in turn retains YAP in the cytoplasm^17^. Moreover, the subsequent phosphorylation of YAP by casein kinase 1δ/ɛ triggers the recruitment of a SKP1-CUL1-F-box protein (SCF) complex, SCF^β-TRCP^, which promotes YAP ubiquitination and degradation under high cell density conditions^18^. Despite the well-established role of Hippo signaling in the regulation of YAP, recent genetic evidence shows that mouse Yap Ser112 (equivalent to Ser127 of human YAP) phosphorylation is dispensable for normal development^19^. Whether additional mechanisms regulate YAP’s subcellular localization and activity remains to be revealed.

In this study, we discovered that YAP undergoes K63-linked polyubiquitination and that this post-translational modification promotes YAP nuclear localization and activity. Furthermore, we identified SKP2 as the E3 ligase that mediates this non-proteolytic ubiquitination, and identified OTUD1 as the deubiquitinating enzyme (DUB) that antagonizes K63-linked ubiquitination and nuclear localization of YAP. These findings provide fresh insights into the regulation of YAP.

## Results

### K63 ubiquitination activates YAP and controls its localization

**Fig. 1:**
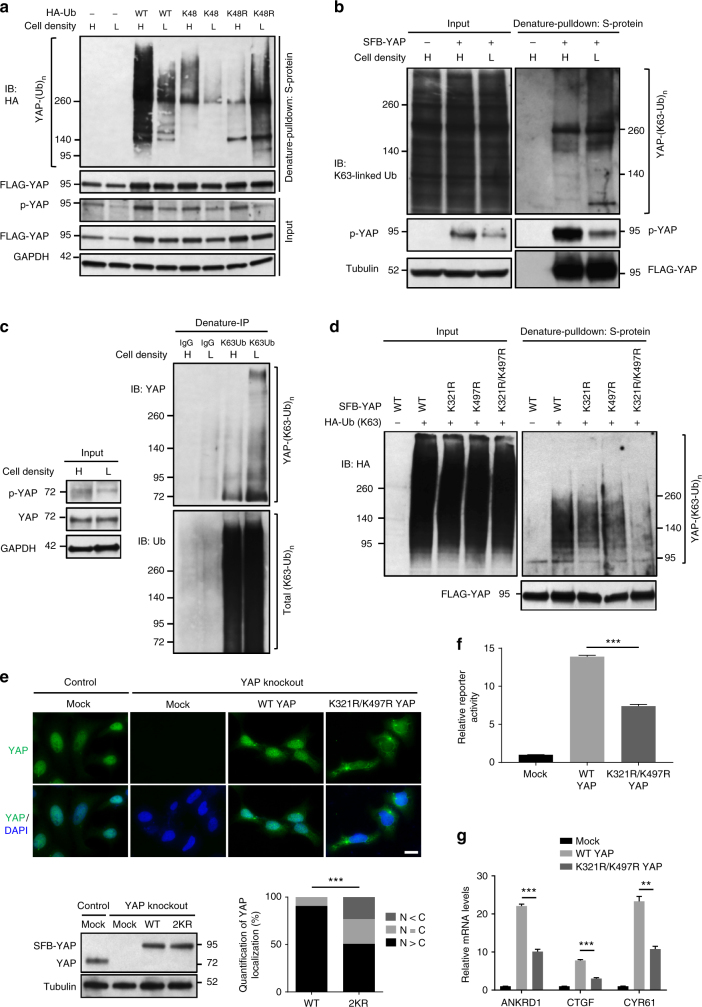
K63-linked ubiquitination promotes YAP nuclear localization and activity. **a** HEK293T cells were transfected with SFB-YAP and HA-ubiquitin (wild-type, K48 or K48R) and then subjected to a pulldown assay with S-protein beads and immunoblotting with antibodies against HA and FLAG. H: high density; L: low density. **b** HEK293A cells were stably transfected with SFB-YAP and then subjected to a pulldown assay with S-protein beads and immunoblotting with antibodies against K63-linkage-specific polyubiquitin, p-YAP (S127) and FLAG. H: high density; L: low density. **c** Total K63-linkage specific ubiquitinated proteins in MCF10A cells were immunoprecipitated by a K63-linked polyubiquitin-specific antibody, followed by immunoblotting with antibodies against YAP and ubiquitin. H: high density; L: low density. **d** HEK293T cells were transfected with SFB-YAP (wild-type, K321R, K497R, or K321R/K497R) and the K63-specific mutant of HA-ubiquitin and then subjected to a pulldown assay with S-protein beads and immunoblotting with antibodies against HA and FLAG. **e** Upper panel: YAP knockout HEK293A cells (generated by CRISPR-Cas9) were transfected with SFB-YAP (wild-type or K321R/K497R) and immunostained with a YAP-specific antibody (green). DAPI (blue) was used to stain DNA. Lower panel: immunoblotting of YAP (left) and quantification of immunofluorescence data (right). Statistical significance was determined by a chi-square test. *P < 0.05; **P < 0.01; ***P < 0.001. Scale bar, 20 μm. **f** Luciferase activity in HEK293T cells co-transfected with SFB-YAP (wild-type or K321R/K497R), an 8×GTIIC luciferase reporter and a TK-Renilla luciferase reporter. *n* = 3 biological replicates. **g** qPCR of *ANKRD1*, *CTGF*, and *CYR61* in cells described in **f**. *n* = 3 biological replicates. Error bars in **f** and **g** are s.e.m. Statistical significance was determined by a two-tailed, unpaired Student’s *t*-test. *P < 0.05; **P < 0.01; ***P < 0.001

To date, whether YAP is regulated by non-proteolytic ubiquitination is unknown. Ubiquitin contains seven lysine (K) residues. While lysine 63 (K63)-linked polyubiquitin chains modulate protein activity, localization and its interaction with other proteins, non-K63 polyubiquitin linkages, particularly K48-linked ubiquitin chains, target proteins for proteasomal degradation^20–22^. Previous studies demonstrated that high cell density activates Hippo signaling leading to phosphorylation and cytoplasmic retention of YAP, and that this condition may also induce proteolytic ubiquitination of YAP by the SCF^β-TRCP^ complex^18,23^. Consistent with these reports, we observed that HEK293T cells cultured at low density showed lower levels of Ser127 phosphorylation, total ubiquitination, and K48-linked polyubiquitination of SFB (S-protein, FLAG, and streptavidin-binding peptide)-tagged YAP, compared with cells at high density (Fig. 1a and Supplementary Fig. 1a). In contrast, using a K48R mutant of ubiquitin, we found that low cell density led to a marked increase in non-K48-linked polyubiquitination of YAP (Fig. 1a). Moreover, using an antibody against K63-linkage specific polyubiquitin^24^, we observed upregulation of K63-linked polyubiquitination of YAP in HEK293A cells cultured at low density (Fig. 1b). We also used this antibody to pull down all K63-linkage specific ubiquitinated proteins from MCF10A cells, and more endogenous YAP was pulled down from low-density cell culture (Fig. 1c). Taken together, these results suggest that K63-linked ubiquitination is associated with active YAP.

Human YAP protein contains 14 lysines. To identify K63-linked ubiquitination sites on YAP, we mutagenized each lysine to arginine and transfected each YAP mutant into HEK293T cells along with a ubiquitin mutant that contains only a single lysine, K63, with all six other lysines mutated to arginine. We found that mutation of two evolutionarily conserved lysine residues of YAP, K321, and K497, consistently led to a pronounced decrease in K63-linked ubiquitination (Supplementary Fig. 1b, c). Mutation of both lysine residues reduced this ubiquitination more drastically (Fig. 1d). Furthermore, we found that the K321-specfic and K497-specific mutants of YAP (i.e., the K321-specific YAP mutant contains only a single lysine, K321, with all 13 other lysines mutated to arginine), but not the all-KR mutant and the K76-specific, K252/K254-specific or K342-specific mutant of YAP, showed K63-linkage specific ubiquitination (Supplementary Fig. 1d). Therefore, K321 and K497 are K63-linked ubiquitination sites on YAP.

Next, we transfected YAP knockout HEK293A cells (generated by the CRISPR-Cas9 approach) with wild-type YAP or the K321R/K497R double mutant. At low cell density, wild-type YAP was predominantly localized in the nucleus in most cells, whereas the K321R/K497R mutant showed substantial cytoplasmic localization, as gauged by immunofluorescent staining (Fig. 1e). Moreover, compared with HEK293T cells transfected with wild-type YAP, cells transfected with the K321R/K497R mutant exhibited lower activity of a YAP-TEAD luciferase reporter (the 8×GTIIC luciferase reporter containing tandem TEAD-binding sites)^25^ and lower mRNA levels of previously reported classical YAP target genes, *ANKRD1*, *CTGF*, and *CYR61* (Fig. 1f, g and Supplementary Fig. 1e). Collectively, these results suggest that K63-linkage specific ubiquitination of YAP is associated with its nuclear localization and activity and is mainly mediated by K321 and K497.

### SKP2 binds and ubiquitinates YAP via K63-specific linkage

**Fig. 2:**
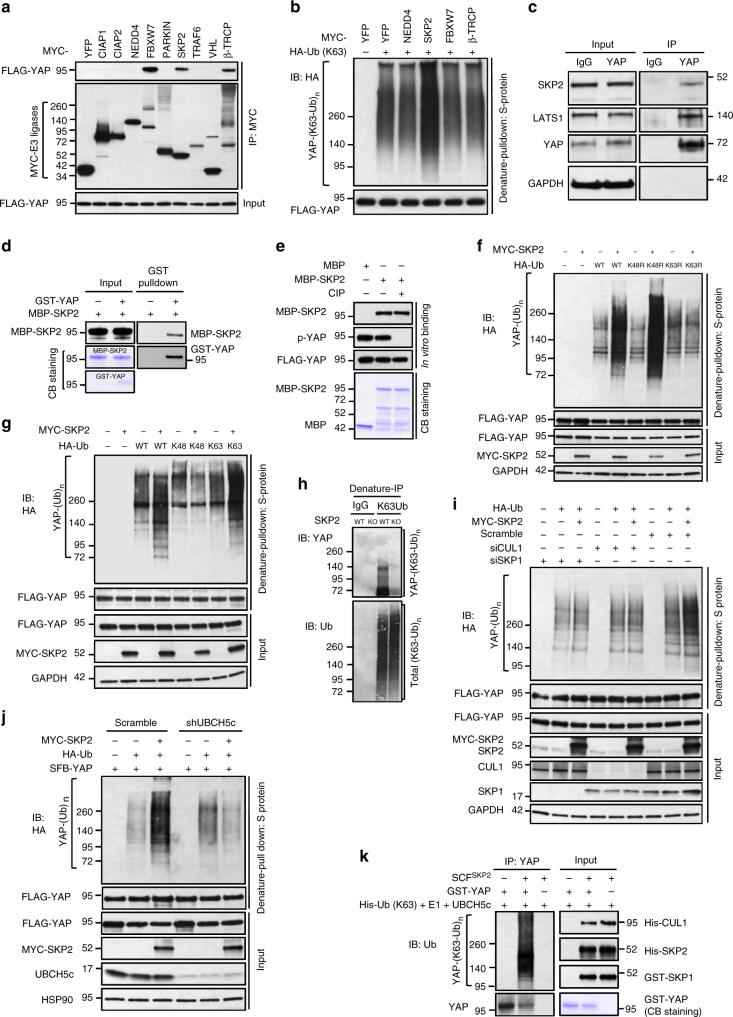
SKP2 interacts with YAP and induces its K63-linked polyubiquitination. **a** The HEK293T SFB-YAP stable cell line was transfected with MYC-tagged E3 ligases, followed by immunoprecipitation with anti-MYC beads and immunoblotting with antibodies against FLAG and MYC. **b** The HEK293T SFB-YAP stable cell line was co-transfected with the K63-specific mutant of HA-ubiquitin and the indicated E3 ligase, followed by pulldown with S-protein beads and immunoblotting with antibodies against HA and FLAG. **c** Co-immunoprecipitation of endogenous SKP2 with endogenous YAP. **d** In vitro binding of bacterially purified MBP-SKP2 to in vitro translated and purified GST-YAP. **e** In vitro binding of bacterially purified MBP-SKP2 to SFB-YAP purified from the HEK293T SFB-YAP stable cell line. MBP-SKP2 or MBP protein was incubated with SFB-YAP protein with or without CIP treatment at 37 °C for 1 h, followed by immunoblotting with antibodies against SKP2, p-YAP (S127), and FLAG. **f** The HEK293T SFB-YAP stable cell line was transfected with HA-ubiquitin (wild-type, K48R or K63R) and MYC-SKP2, followed by pulldown with S-protein beads and immunoblotting with antibodies against HA and FLAG. **g** The HEK293T SFB-YAP stable cell line was transfected with HA-ubiquitin (wild-type, K48-specific or K63-specific) and MYC-SKP2, followed by pulldown with S-protein beads and immunoblotting with antibodies against HA and FLAG. **h** Total K63-linkage specific ubiquitinated proteins in control or SKP2 knockout HEK293A cells (generated by CRISPR-Cas9) were immunoprecipitated by a K63-linked polyubiquitin-specific antibody, followed by immunoblotting with antibodies against YAP and ubiquitin. **i** siRNA targeting *CUL1* or *SKP1* was transfected into the HEK293T SFB-YAP stable cell line. Forty-eight hours after siRNA transfection, cells were transfected with HA-ubiquitin and MYC-SKP2, followed by pulldown with S-protein beads and immunoblotting with antibodies against HA and FLAG. **j** The HEK293T UBCH5c shRNA stable cell line was transfected with SFB-YAP and HA-ubiquitin with or without MYC-SKP2, followed by pulldown with S-protein beads and immunoblotting with antibodies against HA and FLAG. **k** Purified GST-YAP protein was incubated with ATP, E1, UBCH5c, and the K63-specific mutant of His-ubiquitin with or without the SCF^SKP2^ complex, followed by immunoprecipitation with a YAP-specific antibody and immunoblotting with antibodies against ubiquitin and YAP

To identify the ubiquitin ligase (E3) that mediates K63-linked ubiquitination of YAP, we screened a panel of nine E3 ligases, eight of which have been implicated in K63-linked polyubiquitination, for their interaction with YAP and effect on YAP activity; β-TRCP, which targets YAP for degradation^18^, was included as a control. We transfected each MYC-tagged E3 ligase into HEK293T cells that were stably transfected with SFB-tagged YAP. Immunoblotting assays showed that SFB-YAP could be detected on anti-MYC beads conjugated with FBXW7, SKP2, or β-TRCP, but not the other six E3 ligases (Fig. 2a). FBXW7 has been shown to promote both K48-linked (proteolytic) and K63-linked (non-proteolytic) ubiquitination, and both β-TRCP and FBXW7 have been shown to ubiquitinate and degrade YAP^18,26,27^. In our study, all three YAP-interacting E3 ligases (FBXW7, SKP2, and β-TRCP), but not NEDD4, promoted YAP ubiquitination (Supplementary Fig. 2a); however, SKP2 was the only one that increased K63-linked ubiquitination of YAP (Fig. 2b and Supplementary Fig. 2b). Moreover, FBXW7 and β-TRCP decreased, while SKP2 increased YAP reporter activity (Supplementary Fig. 2c).

We further confirmed the interaction between SKP2 and YAP in vivo and in vitro. Endogenous YAP was immunoprecipitated from HEK293A cells, and endogenous SKP2 was detected with a SKP2-specific antibody (Fig. 2c). In addition, MBP (maltose binding protein)-tagged SKP2 purified from bacteria was pulled down by in vitro translated and purified GST (glutathione S-transferase)-tagged YAP, but not by GST, in an in vitro binding assay (Fig. 2d), revealing that SKP2 can directly interact with YAP. It is known that SKP2 recognizes certain substrates (such as p27)^28^ in a phosphorylation-dependent manner. However, calf intestinal alkaline phosphatase (CIP) treatment did not affect the binding of SFB-YAP (which was purified from HEK293T cells and was phosphorylated without CIP treatment) to purified MBP-SKP2 protein (Fig. 2e), suggesting that SKP2 binds YAP in a YAP phosphorylation-independent manner. While the F-box domain is responsible for mediating the interaction of SKP2 to SKP1, the leucine-rich repeats of SKP2 are known to mediate substrate binding^29^. We found that both full-length SKP2 and the F-box deletion mutant (containing the N-terminal region and the C-terminal leucine-rich repeats) could be pulled down by SFB-YAP, whereas the SKP2 fragment lacking the leucine-rich repeats could not (Supplementary Fig. 2d, e), suggesting that the leucine-rich repeats of SKP2 mediate the SKP2-YAP interaction.

We also used the lysine mutants of ubiquitin to validate the polyubiquitin chain linkage specificity. Compared with control cells, SKP2-overexpressing HEK293T cells transfected with either wild-type ubiquitin or its K48R mutant, but not those transfected with the K63R mutant, had much higher levels of YAP polyubiquitination (Fig. 2f and Supplementary Fig. 2f). Moreover, using ubiquitin mutants that are only capable of forming either K48 or K63 linkage, we found that SKP2 specifically induced K63-linked but not K48-linked ubiquitination of YAP (Fig. 2g and Supplementary Fig. 2g). Conversely, knockdown of SKP2 reduced non-K48-linked but not non-K63-linked polyubiquitination of YAP (Supplementary Fig. 2h). Importantly, K63-linked ubiquitination (from endogenous ubiquitin) of endogenous YAP was abolished in SKP2 knockout HEK293A cells generated by CRISPR-Cas9 (Fig. 2h and Supplementary Fig. 2i). These findings further corroborated that SKP2 promotes K63-linked ubiquitination of YAP.

### The SCF^SKP2^ complex confers K63 ubiquitination of YAP

The F-box protein SKP2 is part of an SCF complex that forms a ‘‘super-enzyme’’ together with a ubiquitin-conjugating enzyme (E2)^29–31^. The cullin subunit CUL1 acts as a scaffold that interacts with the adaptor protein SKP1 at the amino terminus and interacts with a RING-finger protein RBX1 at the carboxyl terminus. While RBX1 recruits an E2, SKP1 interacts with SKP2, which in turn binds the substrate. Substrate recognition also requires the accessory protein CKS1^32^. To determine the dependence of SKP2-induced YAP ubiquitination on the SCF complex, we used siRNA to knock down the components of this complex. Notably, silencing of either CUL1, SKP1, or RBX1 abrogated SKP2’s effect on YAP ubiquitination (Fig. 2i and Supplementary Fig. 2j, k), suggesting that this effect is SCF-dependent.

Unlike HECT-type E3 ligases that form covalent intermediates by conjugating with ubiquitin before transferring it to the substrate, SKP2 mediates the transfer of ubiquitin directly from the E2-ubiquitin conjugate to the substrate. Several E2 enzymes, including UBC3^28^ and UBCH5^30,32^, have been shown to work together with the SCF^SKP2^ complex. UBCH5 is considered promiscuous and can catalyze the formation of multiple ubiquitin linkage types, including K63-specific ubiquitin linkage^33,34^. Consistent with this notion, SKP2 has been shown to promote proteolytic ubiquitination of p27^28^ and non-proteolytic, K63-linked ubiquitination of AKT^35^. In the present study, SKP2-induced ubiquitination of YAP was abolished upon knockdown of UBCH5c (Fig. 2j and Supplementary Fig. 2l), suggesting that this ubiquitination is dependent on UBCH5c.

We then incubated purified GST-YAP, E1 enzyme, UBCH5c and the K63-specific mutant of His-ubiquitin in a cell-free system. In the presence of the purified SCF^SKP2^ complex containing CUL1, SKP1, SKP2, RBX1, and CKS1, YAP was robustly polyubiquitinated in vitro via K63-specific ubiquitin linkage (Fig. 2k), suggesting that YAP is a substrate of the SCF^SKP2^ complex.

### SKP2 induces YAP nuclear localization and activity

**Fig. 3:**
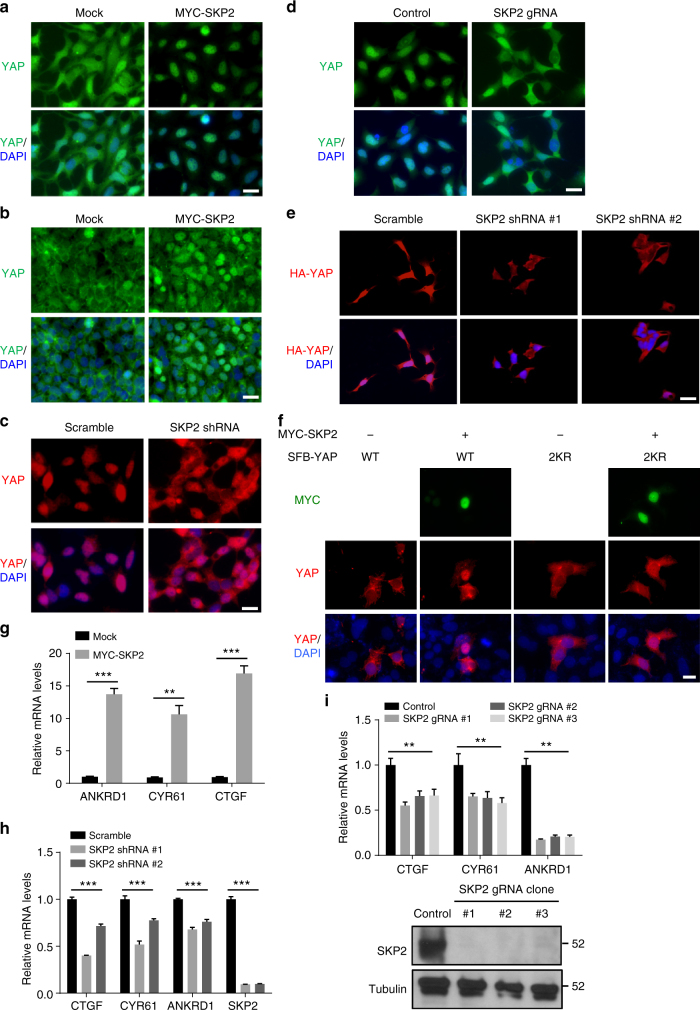
SKP2 promotes nuclear localization and transcriptional activity of YAP. **a**, **b** HEK293A cells were stably transfected with MYC-SKP2, cultured at medium (**a**) or high (**b**) density, and immunostained with a YAP-specific antibody (green). DAPI (blue) was used to stain DNA. Scale bar, 20 μm. **c**, **d** HEK293A cells expressing SKP2 shRNA (**c**) or gRNA (**d**) were immunostained with a YAP-specific antibody (red in **c**; green in **d**). DAPI (blue) was used to stain DNA. Scale bar, 20 μm. **e** HEK293A cells were stably infected with HA-FLAG-YAP and SKP2 shRNA and immunostained with an HA-specific antibody (red). DAPI (blue) was used to stain DNA. Scale bar, 20 μm. **f** YAP knockout HEK293A cells (generated by CRISPR-Cas9) were transfected with SFB-YAP (WT or 2KR) with or without MYC-SKP2, and immunostained with antibodies against YAP (red) and MYC (green). DAPI (blue) was used to stain DNA. Scale bar, 20 μm. **g**–**i** qPCR of *ANKRD1*, *CYR61*, and *CTGF* in HEK293A cells expressing MYC-SKP2 (**g**), SKP2 shRNA (**h**) or SKP2 gRNA (**i**, upper panel). Lower panel in **i**: immunoblotting of SKP2 and α-tubulin in three independent SKP2 knockout HEK293A clones generated by CRISPR-Cas9. *n* = 3 biological replicates. Error bars in **g**–**i** are s.e.m. Statistical significance was determined by a two-tailed, unpaired Student’s *t*-test. *P < 0.05; **P < 0.01; ***P < 0.001

During the E3 ligase screen, SKP2 overexpression substantially increased YAP-TEAD reporter activity (Supplementary Fig. 2c). We asked whether SKP2 regulates the subcellular localization of YAP. In HEK293A cells, endogenous YAP protein was localized in both the cytoplasm and the nucleus at medium cell density (Fig. 3a and Supplementary Fig. 3a) and localized mainly in the cytoplasm at high cell density (Fig. 3b and Supplementary Fig. 3b); under both conditions, ectopic expression of SKP2 led to nuclear translocation of YAP (Fig. 3a, b and Supplementary Fig. 3a, b). At low cell density, endogenous YAP protein was predominantly localized in the nucleus, and either shRNA-mediated knockdown (Fig. 3c and Supplementary Fig. 3c) or gRNA-mediated knockout (Fig. 3d and Supplementary Fig. 3d) of SKP2 resulted in cytoplasmic translocation of YAP. SKP2 depletion also had a similar effect on stably overexpressed YAP protein (Fig. 3e and Supplementary Fig. 3e). These data suggest that SKP2 promotes nuclear localization of YAP. Interestingly, compared with wild-type YAP, the K321R/K497R (2KR) mutant showed less nuclear enrichment upon SKP2 overexpression (Fig. 3f).

Consistent with the effects on YAP localization and reporter activity, SKP2 overexpression upregulated the mRNA levels of the transcriptional target genes of YAP, including *ANKRD1*, *CYR61*, and *CTGF* (Fig. 3g). Moreover, knockdown of SKP2 by two independent shRNAs (Fig. 3h) or CRISPR-Cas9-mediated knockout of SKP2 (Fig. 3i; three independent clones were used) significantly decreased the expression levels of these genes. We conclude from these experiments that SKP2 activates YAP through non-proteolytic ubiquitination and nuclear translocation.

### OTUD1 binds YAP and reverses its K63 ubiquitination

**Fig. 4:**
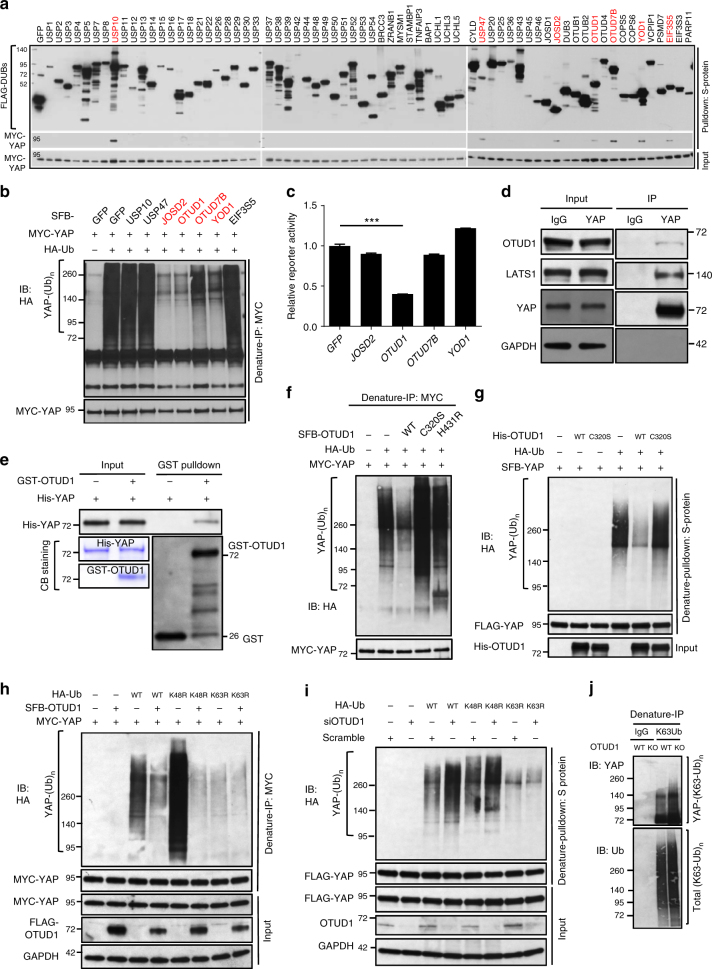
OTUD1 interacts with YAP and antagonizes its K63-linked ubiquitination. **a** SFB-tagged DUBs were co-transfected with MYC-YAP into HEK293T cells, followed by pulldown with S-protein beads and immunoblotting with antibodies against FLAG and MYC. **b** Seven SFB-DUBs were co-transfected with MYC-YAP and HA-ubiquitin into HEK293T cells, followed by immunoprecipitation with anti-MYC beads and immunoblotting with antibodies against HA and MYC. **c** The HEK293T SFB-YAP stable cell line was transfected with four YAP-interacting DUBs along with an 8× GTIIC luciferase reporter and a TK-Renilla luciferase reporter. Reporter activity was measured 48 h after transfection. Error bars are s.e.m. Statistical significance was determined by a two-tailed, unpaired Student’s *t*-test. *P < 0.05; **P < 0.01; ***P < 0.001. *n* = 3 biological replicates. **d** Co-immunoprecipitation of endogenous OTUD1 with endogenous YAP. **e** In vitro binding of purified GST-OTUD1 to purified His-YAP. **f** HEK293T cells were co-transfected with SFB-OTUD1 (wild-type, C320S or H431R), HA-ubiquitin and MYC-YAP, followed by immunoprecipitation with anti-MYC beads and immunoblotting with antibodies against HA and MYC. **g** Ubiquitinated SFB-YAP was purified with S-protein beads and incubated with His-OTUD1 (wild-type or C320S) purified from bacteria. After the in vitro deubiquitination reaction, the bound proteins were eluted by boiling in Laemmli sample buffer and immunoblotted with antibodies against HA, FLAG, and OTUD1. **h** HEK293T cells were co-transfected with SFB-OTUD1, HA-ubiquitin (wild-type, K48R or K63R) and MYC-YAP, followed by immunoprecipitation with anti-MYC beads and immunoblotting with antibodies against HA and MYC. **i** siRNA targeting *OTUD1* was transfected into the HEK293T SFB-YAP stable cell line. Forty-eight hours later, cells were transfected with HA-ubiquitin (wild-type, K48R or K63R), followed by pulldown with S-protein beads and immunoblotting with antibodies against HA and FLAG. **j** Total K63-linkage specific ubiquitinated proteins in control or OTUD1 knockout HEK293A cells (generated by CRISPR-Cas9) were immunoprecipitated by a K63-linked polyubiquitin-specific antibody, followed by immunoblotting with antibodies against YAP and ubiquitin

Humans express approximately 80 functional DUBs^36^. Expanding on our previous study^37^, we constructed an open-reading-frame (ORF) library consisting of 68 human DUBs that were individually fused to the triple-epitope tag SFB. To identify YAP-interacting deubiquitinases, we co-transfected each of the 68 SFB-tagged DUBs with MYC-tagged YAP into HEK293T cells. Immunoblotting assays showed that MYC-YAP could be detected on S-protein beads conjugated with USP10, USP47, JOSD2, OTUD1, OTUD7B, YOD1, or EIF3S5 (Fig. 4a).

To determine the effects of these seven YAP-interacting DUBs on YAP protein level, ubiquitination and activity, we overexpressed each of them in HEK293T cells. Whereas none of these seven DUBs had a substantial effect on YAP protein level (Supplementary Fig. 4a), four of these DUBs—JOSD2, OTUD1, OTUD7B, and YOD1—led to a pronounced reduction of YAP polyubiquitination in HEK293T cells (Fig. 4b and Supplementary Fig. 4b). Using both the K63-specific mutant and the K63R mutant of ubiquitin, we found that of these four YAP-interacting DUBs, only OTUD1 clearly reduced K63-linked ubiquitination of YAP (Supplementary Fig. 4c, d), while the other three DUBs (JOSD2, OTUD7B, and YOD1) substantially reduced non-K63-linked ubiquitination of YAP (Supplementary Fig. 4d). Moreover, of these four candidate DUBs, OTUD1 was the only one that markedly decreased YAP-TEAD reporter activity in HEK293T cells stably transfected with SFB-YAP (Fig. 4c and Supplementary Fig. 4e).

We further validated the interaction between OTUD1 and YAP in vivo and in vitro. Endogenous YAP was immunoprecipitated from MCF10A cell lysates, and endogenous OTUD1 was detected with an OTUD1-specific antibody (Fig. 4d). Moreover, His-YAP protein purified from bacteria could be pulled down by GST-OTUD1, but not by GST, in an in vitro binding assay (Fig. 4e), suggesting that OTUD1 directly interacts with YAP.

The *OTUD1* gene is located on chromosome 10p12^38^ and encodes an ovarian tumor protease (OTU) domain-containing cysteine protease that preferentially cleaves K63-linked di-ubiquitin in vitro^39^. However, specific protein substrates whose K63-linked polyubiquitination is inhibited by OTUD1 remain unknown. We reasoned that OTUD1 is a K63-linkage specific DUB for YAP. In HEK293T cells, overexpression of wild-type OTUD1, but not its catalytically inactive mutants, C320S and H431R^39^, reduced the polyubiquitination of YAP (Fig. 4f and Supplementary Fig. 4f). To further determine whether OTUD1 directly deubiquitinates YAP, we incubated purified OTUD1 and ubiquitinated YAP in a cell-free system. Wild-type OTUD1, but not the C320S mutant, markedly decreased YAP polyubiquitination in vitro (Fig. 4g and Supplementary Fig. 4g), suggesting that YAP is a substrate of OTUD1. Moreover, upon overexpression of OTUD1, the polyubiquitination of YAP was downregulated in HEK293T cells transfected with wild-type ubiquitin or its K48R mutant, but not in cells transfected with the K63R mutant (Fig. 4h and Supplementary Fig. 4h). Conversely, silencing of OTUD1 by siRNA increased non-K48-linked but not non-K63-linked polyubiquitination of YAP (Fig. 4i and Supplementary Fig. 4i). Importantly, K63-linked ubiquitination (from endogenous ubiquitin) of endogenous YAP was upregulated in OTUD1 knockout HEK293A cells generated by CRISPR-Cas9 (Fig. 4j and Supplementary Fig. 4j). Taken together, these results suggest that OTUD1 specifically reverses non-proteolytic, K63-linked ubiquitination of YAP.

### OTUD1 retains YAP in the cytoplasm and inhibits its activity

**Fig. 5:**
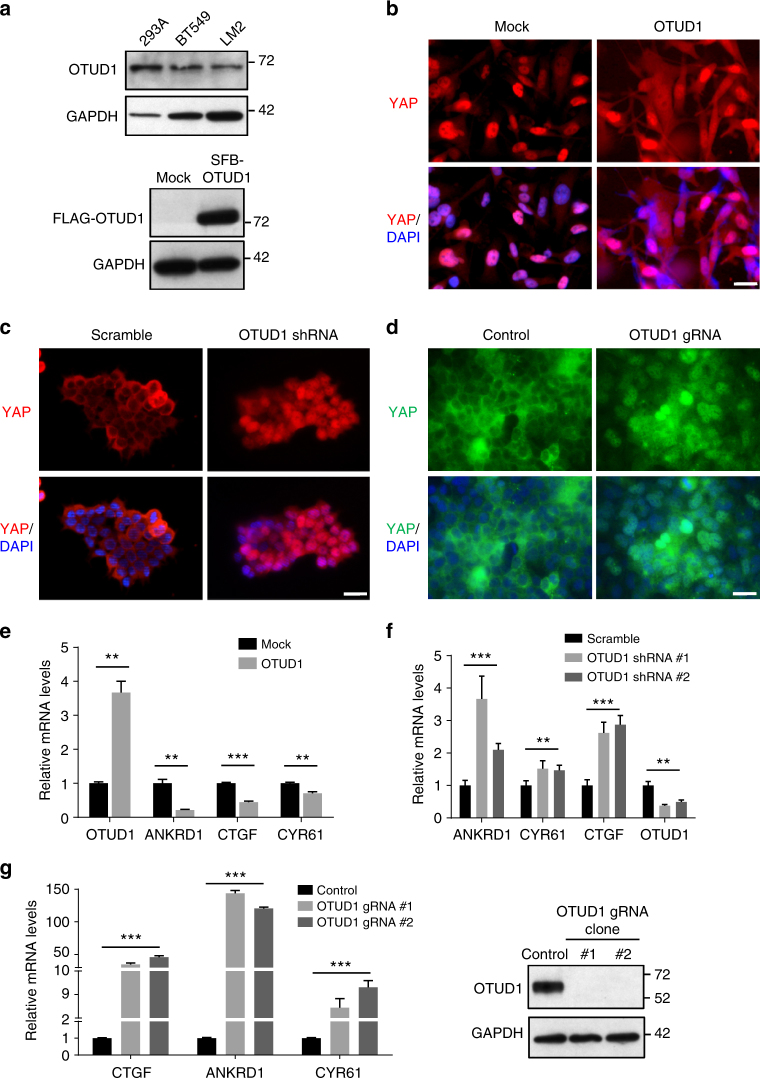
OTUD1 inhibits nuclear localization and transcriptional activity of YAP. **a** Upper panel: immunoblotting of OTUD1 and GAPDH in HEK293A, BT549, and LM2 cell lines. Lower panel: immunoblotting of FLAG-OTUD1 and GAPDH in LM2 cells stably transfected with SFB-OTUD1. **b** LM2 cells stably overexpressing OTUD1 were immunostained with a YAP-specific antibody (red). DAPI (blue) was used to stain DNA. Scale bar, 20 μm. **c**, **d** HEK293A cells expressing OTUD1 shRNA (**c**) or gRNA (**d**) were immunostained with a YAP-specific antibody (red in **c**; green in **d**). DAPI (blue) was used to stain DNA. Scale bar, 20 μm. **e**–**g** qPCR of *ANKRD1*, *CTGF*, and *CYR61* in BT549 cells expressing HA-OTUD1 (**e**) and in HEK293A cells expressing OTUD1 shRNA (**f**) or OTUD1 gRNA (**g**, left panel). Right panel in **g**: immunoblotting of OTUD1 and GAPDH in two independent OTUD1 knockout HEK293A clones generated by CRISPR-Cas9. *n* = 3 biological replicates. Error bars in **e**–**g** are s.e.m. Statistical significance was determined by a two-tailed, unpaired Student’s *t*-test. *P < 0.05; **P < 0.01; ***P < 0.001

Based on the reporter assay (Fig. 4c and Supplementary Fig. 4e), OTUD1 regulates YAP activity but not its expression level, prompting us to determine whether OTUD1 affects YAP localization. Compared with HEK293A cells, triple-negative breast cancer cell lines such as BT549 and the LM2 lung-metastatic subline of MDA-MB-231 cells showed much lower levels of OTUD1 protein (Fig. 5a). Notably, overexpression of OTUD1 in LM2 cells led to cytoplasmic translocation of endogenous YAP at low density (Fig. 5a, b and Supplementary Fig. 5a). On the other hand, depletion of OTUD1 by either shRNA-mediated knockdown (Fig. 5c and Supplementary Fig. 5b) or gRNA-mediated knockout (Fig. 5d and Supplementary Fig. 5c) led to nuclear translocation of endogenous YAP in HEK293A cells at high density. Moreover, the expression levels of classical YAP target genes, *ANKRD1*, *CTGF* and *CYR61*, were downregulated by overexpression of OTUD1 (Fig. 5e) and upregulated by knockdown (Fig. 5f) or knockout (Fig. 5g) of OTUD1. Therefore, OTUD1 is a negative regulator of YAP nuclear localization and activity.

### K63 ubiquitination of YAP is independent of Hippo signaling

**Fig. 6:**
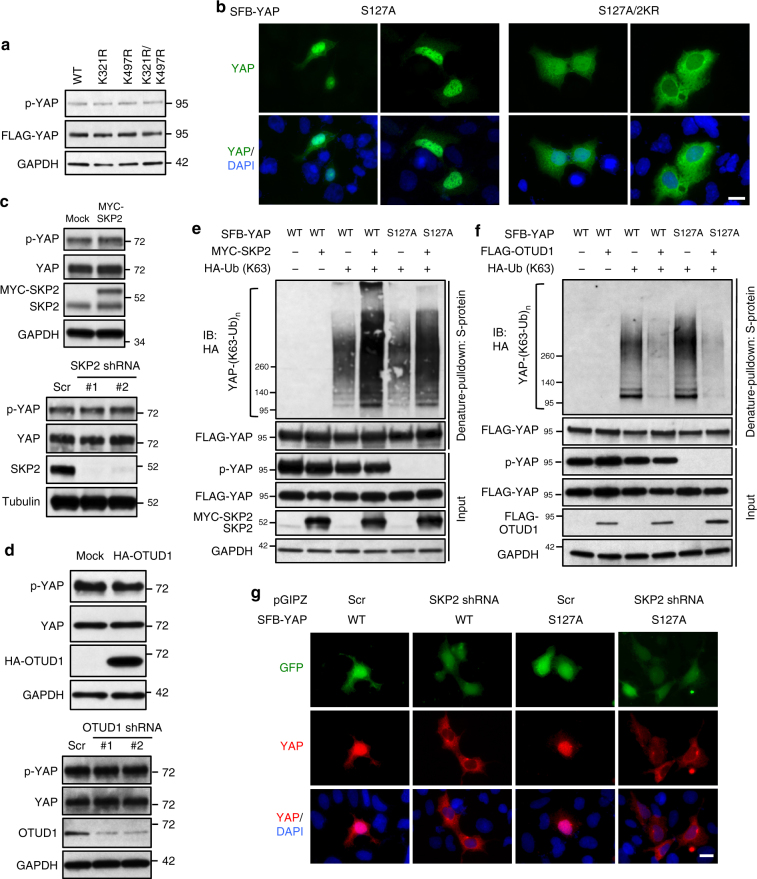
K63-linked ubiquitination of YAP is independent of Hippo signaling-mediated YAP phosphorylation. **a** Immunoblotting of p-YAP (S127), FLAG-YAP, and GAPDH in HEK293T cells transfected with SFB-YAP (wild-type, K321R, K497R, or K321R/K497R). **b** YAP knockout HEK293A cells (generated by CRISPR-Cas9) were transfected with SFB-YAP (S127A or S127A/K321R/K497R) and immunostained with a YAP-specific antibody (green). DAPI (blue) was used to stain DNA. Two different fields are shown. Scale bar, 20 μm. **c** Immunoblotting of p-YAP (S127), YAP, and SKP2 in HEK293A cells stably expressing MYC-SKP2 (upper panel) or SKP2 shRNA ( lower panel). Scr: a scramble control. **d** Immunoblotting of p-YAP (S127), YAP, OTUD1, and GAPDH in HEK293A cells stably transfected with HA-OTUD1 (upper panel) or OTUD1 shRNA (lower panel). Scr: a scramble control. **e** HEK293T cells were co-transfected with SFB-YAP (wild-type or S127A), MYC-SKP2 and the K63-specific mutant of HA-ubiquitin and then subjected to a pulldown assay with S-protein beads and immunoblotting with antibodies against HA and FLAG. **f** HEK293T cells were co-transfected SFB-YAP (wild-type or S127A), FLAG-OTUD1 and the K63-specific mutant of HA-ubiquitin and then subjected to a pulldown assay with S-protein beads and immunoblotting with antibodies against HA and FLAG. **g** YAP knockout HEK293A cells (generated by CRISPR-Cas9) were transfected with SFB-YAP (WT or S127A) with or without SKP2 shRNA (on a GFP-positive pGIPZ vector), and immunostained with a YAP-specific antibody (red). DAPI (blue) was used to stain DNA. Scr: a scramble control. Scale bar, 20 μm

We asked whether the regulation of YAP by K63-linked ubiquitination is Hippo-dependent. Compared with HEK293T cells transfected with wild-type YAP, cells transfected with the K321R/K497R mutant showed the same levels of total YAP protein and S127-phosphorylated YAP (Fig. 6a). As expected, the S127A mutant of YAP was predominantly localized in the nucleus in most cells, whereas mutation of K321 and K497 (2KR) induced cytoplasmic localization of the S127A mutant (Fig. 6b and Supplementary Fig. 6a), similar to the effect on wild-type YAP (Fig. 1e), indicating the independence on S127 phosphorylation. Furthermore, neither overexpression nor depletion of SKP2 altered endogenous YAP protein level or its S127 phosphorylation (Fig. 6c). Similar results were obtained with overexpression or depletion of OTUD1 (Fig. 6d). Importantly, similar to the effect on wild-type YAP, K63-linked polyubiquitination of the S127A mutant of YAP was induced by overexpression of SKP2 (Fig. 6e and Supplementary Fig. 6b), and was drastically reduced by overexpression of OTUD1 (Fig. 6f and Supplementary Fig. 6c). Moreover, knockdown of SKP2 caused cytoplasmic translocation of both wild-type YAP and the S127A mutant (Fig. 6g). Collectively, these data corroborate the notion that SKP2 and OTUD1 regulate non-proteolytic ubiquitination of YAP independently of Hippo signaling-mediated YAP phosphorylation.

### SKP2 and K63 ubiquitination promote the YAP–TEAD interaction

**Fig. 7:**
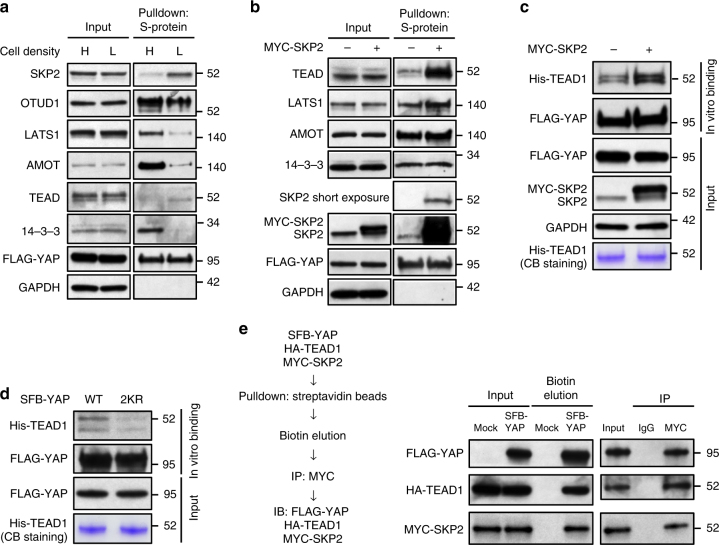
SKP2 and K63-linked ubiquitination enhance the YAP-TEAD interaction. **a** HEK293A cells were stably transfected with SFB-YAP and then subjected to a pulldown assay with S-protein beads and immunoblotting with antibodies against YAP-interacting proteins. H: high density; L: low density. **b** The HEK293A SFB-YAP stable cell line was transfected with MYC-SKP2 and then subjected to a pulldown assay with S-protein beads and immunoblotting with antibodies against YAP-interacting proteins. **c** The HEK293A SFB-YAP stable cell line was transfected with MYC-SKP2. SFB-YAP was pulled down with S-protein beads and then incubated with His-TEAD1 protein purified from bacteria. After in vitro binding, the proteins bound to S-protein beads were eluted by boiling in Laemmli sample buffer and immunoblotted with antibodies against His and FLAG tags. **d** HEK293T cells were transfected with SFB-YAP (WT or the 2KR mutant). SFB-YAP was pulled down with S-protein beads and then incubated with His-TEAD1 protein purified from bacteria. After in vitro binding, the proteins bound to S-protein beads were eluted by boiling in Laemmli sample buffer and immunoblotted with antibodies against His and FLAG tags. **e** SKP2 forms a complex with YAP and TEAD1. HA-TEAD1 and MYC-SKP2 were co-transfected with or without SFB-YAP into HEK293T cells. SFB-YAP was pulled down with streptavidin beads and then eluted with biotin buffer. The eluded proteins were subjected to immunoprecipitation with a MYC-specific antibody to pull down MYC-SKP2 and immunoblotting with antibodies against FLAG, HA, and MYC

K63-linked ubiquitination of YAP was upregulated in cells at low density (Fig. 1c), which prompted us to examine whether cell density can influence the interaction of YAP with SKP2 or OTUD1. At high cell density, YAP showed more interaction with LATS1, AMOT, and 14-3-3, and less interaction with TEAD (Fig. 7a). Consistent with the inverse correlation between cell density and K63-linked YAP ubiquitination, SKP2 exhibited stronger interaction with YAP at low cell density, whereas OTUD1 showed stronger interaction with YAP at high cell density (Fig. 7a).

Next, we sought to address how K63-linked ubiquitination of YAP promotes its nuclear localization and activity. To this end, we examined the interaction between YAP and its binding proteins in SKP2-overexpressing HEK293A and BT549 cells. Compared with control cells, SKP2 overexpression had moderate or no effect on the interaction of YAP with LATS1, AMOT, or 14-3-3, but markedly increased the interaction between YAP and TEAD in both cell lines (Fig. 7b and Supplementary Fig. 7a). The enhanced YAP-TEAD association could be either a cause or a consequence of SKP2-induced nuclear localization of YAP. To distinguish between these two possibilities, we pulled down SFB-tagged YAP protein from control or SKP2-overexpressing HEK293A cells, and incubated it with His-tagged TEAD1 protein purified from bacteria. YAP protein purified from SKP2-overepressing cells showed stronger binding to purified His-TEAD1 protein in vitro (Fig. 7c). These results suggest that SKP2 promotes the YAP-TEAD interaction, which in turn retains YAP in the nucleus. Importantly, mutation of K321 and K497 (2KR) markedly impaired in vitro binding of YAP to purified His-TEAD1 protein (Fig. 7d), suggesting that K63-linked ubiquitination of YAP is critical for its interaction with TEAD. Furthermore, co-immunoprecipitation revealed the interaction of SKP2 with TEAD1 (Supplementary Fig. 7b), and sequential pulldown experiments demonstrated that TEAD1 could be detected in the SKP2-YAP complex (Fig. 7e), suggesting that SKP2, YAP, and TEAD are assembled in a ternary complex.

### K63 ubiquitination activates YAP’s growth-promoting function

**Fig. 8:**
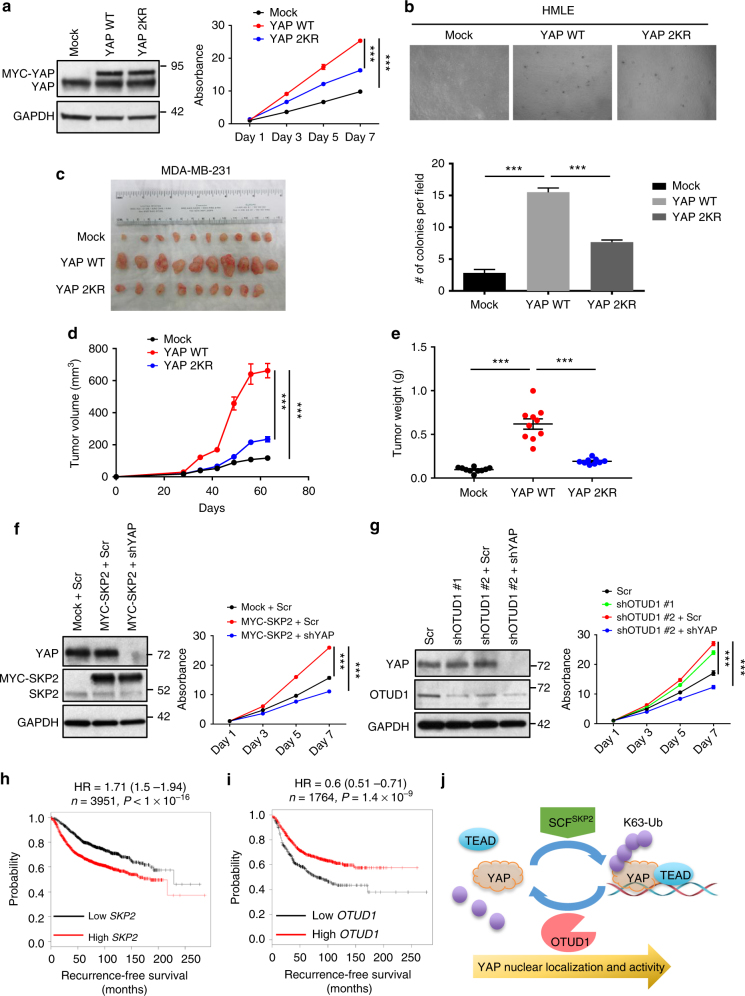
K63-linked ubiquitination of YAP induces its growth-promoting function. **a** Left panel: immunoblotting of YAP and GAPDH in HMLE cells transduced with wild-type (WT) YAP or the K321R/K497R mutant (2KR). Right panel: growth curves. *n* = 5 biological replicates. **b** Images (upper panel) and quantification (lower panel) of soft agar colony formation by the cells described in (**a**). *n* = 3 biological replicates. **c** Endpoint images of tumors dissected from NSG mice with mammary fat pad injection of MDA-MB-231 cells transduced with wild-type (WT) YAP or the K321R/K497R mutant (2KR). *n* = 10 (mock), 10 (YAP WT) and 9 (YAP 2KR) mice per group. **d**, **e** Tumor growth curves (**d**) and tumor weight (at the endpoint, **e**) of NSG mice with mammary fat pad injection of MDA-MB-231 cells transduced with wild-type (WT) YAP or the K321R/K497R mutant (2KR). *n* = 10 (mock), 10 (YAP WT) and 9 (YAP 2KR) mice per group. **f** Left panel: immunoblotting of YAP, SKP2, and GAPDH in BT549 cells transduced with MYC-SKP2 with or without YAP shRNA. Right panel: growth curves. *n* = 5 biological replicates. **g** Left panel: immunoblotting of YAP, OTUD1 and GAPDH in BT549 cells transduced with OTUD1 shRNA with or without YAP shRNA. Right panel: growth curves. *n* = 5 biological replicates. **h**, **i** Kaplan–Meier curves of recurrence-free survival of patients with breast cancer, stratified by *SKP2* (*n* = 3951 patients, probe: 203625_x_at, (**h**) or *OTUD1* (*n* = 1764 patients, probe: 226140_s_at, (**i**) expression levels. Data were obtained from http://kmplot.com/analysis/. Auto-select best cutoff was used in the analysis. **j** Model for the regulation of YAP localization and activity by non-proteolytic ubiquitination. Error bars in **a**, **b**, and **d**–**g** are s.e.m. Statistical significance was determined by a two-tailed, unpaired Student’s *t*-test. *P < 0.05; **P < 0.01; ***P < 0.001

To determine the role of K63-linked ubiquitination in regulating YAP’s function, we ectopically expressed wild-type YAP or the K321R/K497R (2KR) double mutant in HMLE cells, a non-transformed human mammary epithelial cell line. Compared with wild-type YAP, the K63-linked ubiquitination-deficient YAP mutant (2KR) had less ability to induce cell proliferation (Fig. 8a) and anchorage-independent growth (Fig. 8b). Similarly, MDA-MB-231 and BT549 breast cancer cells expressing wild-type YAP proliferated faster than cells expressing the 2KR mutant (Supplementary Fig. 8a, b). Next, we implanted MDA-MB-231 cells expressing either wild-type YAP or the 2KR mutant into the mammary fat pads of NSG (non-obese diabetic; severe combined immunodeficiency; interleukin-2 receptor gamma chain null) mice, and found that the K321R/K497R mutation drastically impaired the tumor-promoting effect of YAP (Fig. 8c–e).

SKP2 is a well-established oncogenic E3 ligase. Previous studies demonstrated that overexpression of SKP2 induced tumor formation while ablation of SKP2 impaired tumorigenesis in xenograft models and genetically engineered mouse models^35,40,41^. OTUD1 is an understudied DUB. Notably, ectopic expression of SKP2 (Fig. 8f) or knockdown of OTUD1 (Fig. 8g) promoted BT549 cell growth, which was reversed by depletion of YAP (Fig. 8f, g), suggesting that YAP mediates, at least in part, the growth-promoting effect of SKP2 overexpression or OTUD1 depletion.

YAP has been shown to promote breast tumorigenesis and metastasis^6,7,42^. Moreover, compared with normal mammary tissues, a higher percentage of breast tumors showed strong nuclear YAP expression^16^. To assess whether the expression of *SKP2* or *OTUD1* is associated with clinical outcomes in breast cancer patients, we performed Kaplan–Meier (KM) plotter analysis of microarray datasets of human mammary tumors^43^. Notably, patients with high levels of *SKP2* or low levels of *OTUD1* in the tumors had shorter recurrence-free survival than patients with low levels of *SKP2* or high levels of *OTUD1* (Fig. 8h, i).

## Discussion

The Hippo pathway effector YAP is a transcriptional co-activator that promotes growth and tumorigenesis. The nuclear localization of YAP is inhibited by the Hippo phosphorylation cascade; however, Hippo-independent regulation of YAP has not been fully unraveled. In the present study, we discovered a previously undescribed mechanism by which K63-linked polyubiquitination of YAP promotes its nuclear localization, transcriptional activity, and growth-promoting function (Fig. 8j). Moreover, we identified the E3 ligase and the DUB that regulate this non-proteolytic ubiquitination without altering YAP protein level and Hippo pathway-mediated phosphorylation of YAP.

SKP2, which belongs to the F-box protein family, induces ubiquitination and degradation of p27 and p21 through the SCF complex^28,44,45^. The SCF^SKP2^ complex has long been believed to promote proteolytic, K48-linked ubiquitination of its substrates, including p27 and p21, with the help of a specific E2 enzyme, such as UBC3, UBC4 or UBC5^31^. However, several reports indicated that SKP2 also regulates non-proteolytic ubiquitination of certain substrates, such as AKT^35^. Our study identified YAP as a substrate of SKP2 and uncovered SKP2’s role in mediating YAP’s non-proteolytic, K63-linked ubiquitination through the SCF complex and UBCH5c, an E2 enzyme that has been shown to promote the formation of K11-, K48-, and K63-linked polyubiquitin chains^46^. Recently, Sun and colleagues identified FBXW7, an F-box protein previously reported to promote proteolytic, K48-linked ubiquitination exclusively, as an E3 ligase that induces K63-linked ubiquitination of XRCC4 through the SCF complex upon exposure to ionizing radiation^47^. Taken together, these findings provide evidence for the emerging role of F-box proteins in mediating K63-linked ubiquitination.

Whereas our study identified SKP2 as a positive regulator of YAP, SKP2 may be regulated by YAP under certain conditions. Recently, *SKP2* was reported as a YAP target gene in human cell lines, but not in mouse cells^48^. Another recent study showed that deletion of *Mst1/2* or *lats1/2*, or transgenic overexpression of *Yap*, led to cytoplasmic translocation of Skp2 protein in mice^49^. Interestingly, a public protein database showed nuclear localization of SKP2 in human cells but cytoplasmic localization of Skp2 in mouse cells (http://www.proteinatlas.org/ENSG00000145604-SKP2/cell). The mechanisms underlying differential transcriptional regulation and subcellular localization of SKP2 between human and mouse cells warrant further investigation.

While the E3 ligase complexes SCF^β-TRCP^ and SCF^FBXW7^ have been shown to ubiquitinate YAP leading to its degradation^18,26,27^, USP9X, the only reported YAP DUB, was recently shown to promote tumor cell survival and chemoresistance through deubiquitination and stabilization of YAP^50^. However, other studies have revealed that USP9X deubiquitinates and stabilizes LATS to suppress tumor growth^51,52^. Thus, the role of USP9X in regulating YAP and tumorigenesis remains controversial. Here, we report that the deubiquitinase OTUD1 specifically reverses non-proteolytic, K63-linked ubiquitination of YAP, leading to its cytoplasmic sequestration and functional inactivation. OTUD1 is an OTU family member that preferentially cleaves K63-linked ubiquitin chains^39^. Our findings identify YAP as a K63-linkage specific OTUD1 substrate. Given the effects of SKP2 and OTUD1 on YAP nuclear localization and activity, future studies should determine whether SKP2 and OTUD1 are modulators of YAP-induced organ growth, tissue regeneration and tumorigenesis, using genetically engineered mouse models.

## Methods

### Cell lines

The HEK293A, HEK293T, MDA-MB-231, LM2, and BT549 cell lines were cultured in Dulbecco’s modified Eagle’s medium (DMEM) supplemented with 10% fetal bovine serum. The MCF10A cell line was cultured in DMEM/F12 medium supplemented with 5% horse serum, 20 ng ml^-1^ epidermal growth factor, 0.5 mg ml^-1^ hydrocortisone, 100 ng ml^-1^ cholera toxin, and 10 μg ml^-1^ insulin. The HMLE cell line was cultured in complete Mammary Epithelial Cell Growth Medium (MEGM from Lonza). The BT549, MCF10A, MDA-MB-231, and HEK293T cell lines were from ATCC. The HEK293A cell line was from Dr. Junjie Chen (MD Anderson Cancer Center, Houston, USA). The HMLE cell line was from Dr. Robert A. Weinberg (Whitehead Institute for Biomedical Research, Cambridge, USA). The LM2 cell line was from Dr. Xiang Zhang (Baylor College of Medicine, Houston, USA). The cell lines used in this study were tested negative for mycoplasma contamination. Short tandem repeat (STR) profiling was done by ATCC and MD Anderson’s Characterized Cell Line Core Facility for cell line authentication.

### Plasmids and siRNA

Nine human E3 ligase ORFs and human YAP and SKP2 ORFs were individually subcloned into the pCDH-MYC vector. Sixty-eight human DUB ORFs were individually subcloned into the pBabe-SFB vector. The His-GST-OTUD1 (#61405), MYC-SKP2 (#19947), and HA-ubiquitin (wild-type: #17608; K48R: #17604; K48: #17605; K63: #17606) constructs were from Addgene. The SFB-YAP, MYC-YAP, and HA-YAP constructs were gifts of Dr. Junjie Chen. The YAP-TEAD luciferase reporter construct was from Dr. Mien-Chie Hung (MD Anderson Cancer Center, Houston, USA). The K63R mutant of HA-ubiquitin, the lysine-to-arginine mutants of SFB-YAP and MYC-YAP, the C320S and H431R mutants of SFB-OTUD1, and the C320S mutant of His-GST-OTUD1 were generated using the QuikChange® kit from Agilent Technologies and validated by sequencing. Xpress-tagged full-length SKP2 and deletion mutants were from Dr. Hui-Kuan Lin’s lab (Wake Forest School of Medicine, Winston-Salem, USA). The UBCH5c shRNA (clone ID: NM_003340.4-849s1c1) and OTUD1 shRNA (clone ID: NM_001145373.2-1401s21c1 and NM_001145373.2-1725s21c1) constructs were from Sigma. The SKP2 shRNA (clone ID: V2LHS_199794 and V2LHS_199552) and YAP shRNA (clone ID: V2LHS_65508) constructs were from Dharmacon. siRNAs (CUL1: EHU070971; SKP1: EHU134251; OTUD1: EHU126001) were from Sigma. The human RBX1 siRNA was synthesized by Sigma and the sequence is 5′-GACTTTCCCTGCTGTTACCTAA-3′.

### CRISPR-Cas9-mediated gene editing

The HEK293A YAP knockout cell line (generated by CRISPR-Cas9) was a gift of Dr. Junjie Chen. For the generation of SKP2 and OTUD1 knockout cell lines, CRISPR-Cas9 constructs from Santa Cruz Biotechnology (SKP2: sc-400534; OTUD1: sc-405431) were used. HEK293A cells were transfected with the gRNA expression vector containing GFP, sorted by GFP and seeded in 96-well plates for single colony isolation. The isolated clones were subjected to sequencing and western blot analysis.

### Viral transduction

Virus-containing supernatant was collected 48 h after co-transfection of the viral vector and packaging plasmids (psPAX2 and pMD2.G) into HEK293T cells, and then added to the target cells. The infected cells were selected with 1 μg ml^-1^ puromycin or 300 μg ml^-1^ hygromycin B.

### Immunoblotting

Western blot analysis was performed with precast gradient gels (Bio-Rad) using standard methods. Briefly, cultured cells were lysed in RIPA buffer (Millipore, 20–188) containing protease inhibitors (Roche) and phosphatase inhibitors (Sigma). Proteins were separated by SDS-PAGE and blotted onto a nitrocellulose membrane (Bio-Rad). Membranes were probed with the specific primary antibodies, followed by peroxidase-conjugated secondary antibodies. The bands were visualized by chemiluminescence (Denville Scientific). Antibodies against YAP (14074, 1:1000), phospho-YAP (4911, 1:500), SKP2 (2652, 1:1000), UBCH5c (4330, 1:500), GST (2624, 1:1000), Angiomotin (43130, 1:1000), TEAD (13295, 1:500), 14-3-3 (8312, 1:500), RBX1 (11922, 1:500), and LATS1 (3477, 1:500) were from Cell Signaling Technology. Antibodies against CUL1 (612040, 1:300), SKP1 (610530, 1:300), and HSP90 (610419, 1:2500) were from BD Biosciences. Antibodies against OTUD1 (HPA038504, 1:100), His (SAB2702218, 1:500), FLAG (F3165, clone M2, 1:5000), and α-tubulin (T5168, 1:3000) were from Sigma. Antibodies against SKP2 (32-3300, 1:500), Xpress (R910-25, 1:1000), and GAPDH (MA5-15738, 1:3000) were from ThermoFisher Scientific. Antibodies against ubiquitin (sc-8017, 1:1000), HA (sc-7392, 1:2000), β-actin (sc-47778, 1:1000), and MYC (sc-40, clone 9E10, 1:2000) were from Santa Cruz Biotechnology. The antibody against K63-linked polyubiquitin (05-1308, 1:500) was from Millipore. The uncropped blots are shown in Supplementary Fig. 9.

### Immunoprecipitation and pulldown assays

Cells were lysed in CHAPS buffer (120 mM NaCl, 20 mM NaF, 1 mM EDTA, 25 mM Tris-HCl, pH 7.5, 0.33% CHAPS) containing protease inhibitors (Roche). For immunoprecipitation of protein complexes, cell extracts were pre-cleared with protein-A/G beads and incubated with a YAP-specific antibody (Cell Signaling Technology, 14074) at 4 °C overnight. For pulldown of SFB-tagged proteins, cell extracts were incubated with S-protein beads (Millipore, 69704) at 4 °C for 2 h. For in vitro binding assays, purified GST-YAP (Novus Biologicals, H00010413-P01) was incubated with purified MBP-SKP2, and purified His-YAP (MyBioSource, MBS717875) was incubated with purified GST-OTUD1, followed by pulldown with Glutathione Sepharose beads (GE healthcare, 17-0756-01).

### Immunofluorescence

Immunofluorescent staining was performed as described previously^42^. Briefly, cells were cultured in chamber slides, fixed with 3.7% formaldehyde and then permeabilized with 0.5% Triton X-100. Cells were then blocked for non-specific binding with goat serum and bovine serum albumin, and incubated with an antibody against YAP (1:200, Cell Signaling Technology, 14729 or 14074), SKP2 (1:100,  ThermoFisher Scientific, 32-3300), HA (1:500, Santa Cruz Biotechnology, sc-7392), MYC (1:500, Santa Cruz Biotechnology, sc-40), or FLAG (1:800, Sigma, F3165) at 4 °C overnight, followed by incubation with Alexa Fluor 594 goat anti-rabbit IgG (1:500, Invitrogen by ThermoFisher Scientific, A11012), Alexa Fluor 594 goat anti-mouse IgG (1:500, Invitrogen by ThermoFisher Scientific, A11005) or Alexa Fluor 488 goat anti-mouse IgG (1:500, Invitrogen by ThermoFisher Scientific, A11001) at 37 °C for 1 h. Cover slips were mounted on slides using antifade mounting medium with DAPI (Vector Laboratories, H-1200). Immunofluorescence images were acquired on a Zeiss Axio Observer Z1 microscope.

### Luciferase reporter assay

The luciferase reporter assay was performed as described previously^42^. Briefly, HEK293T cells were transfected with the indicated plasmid along with an 8× GTIIC luciferase reporter and a TK-Renilla luciferase reporter. Reporter activity was analyzed 48 h after transfection.

### RNA isolation and qPCR

Total RNA was isolated using TRIzol reagent (Invitrogen) and then reverse transcribed with an iScript complementary DNA (cDNA) Synthesis Kit (Bio-Rad). The resulting cDNA was used for real-time PCR using the iTaq Universal SYBR Green Kit (Bio-Rad). β-actin was used as an internal control. Real-time PCR and data collection were performed on a CFX96 instrument (Bio-Rad). The primer sequences are provided in Supplementary Table 1.

### In vivo and in vitro ubiquitination assays

For the in vivo ubiquitination assay, HEK293T cells were harvested 48 hours after transfection with the indicated plasmids. For denaturing, lysates were heated at 95 °C for 5 minutes in the presence of 1% SDS, followed by tenfold dilution with lysis buffer (to 0.1% SDS) and sonication, as described previously^24^. The cell lysates were incubated with S-protein or anti-MYC beads for 2 h, and then the beads were washed with lysis buffer three times and subjected to immunoblotting analysis. For pulldown of total K63-linkage specific ubiquitinated proteins, cells were lysed and subjected to immunoprecipitation with a K63-linked polyubiquitin-specific antibody, followed by immunoblotting with antibodies against YAP and ubiquitin. For the in vitro ubiquitination assay, 1 μg purified GST-YAP protein (Novus Biologicals, H00010413-P01) was incubated with 1 μg purified SCF^SKP2^ complex (Millipore, 23-023) at 37 °C for 3 h in 20 μl of reaction buffer including ATP, E1 (BostonBiochem, ubiquitin conjugation initiation kit, K-995), 1 μM UBCH5c (BostonBiochem, K-980B) and 10 μM K63-specific mutant of His-ubiquitin (BostonBiochem, UM-HK630). The reaction was terminated by EDTA (final concentration, 10 mM). GST-YAP was pulled down by a YAP-specific antibody (Cell Signaling Technology, 14074), followed by immunoblotting with antibodies against ubiquitin (Santa Cruz Biotechnology, sc-8017, 1:500) and YAP (Cell Signaling Technology, 14074, 1:1000).

### In vivo and in vitro deubiquitination assays

For the in vivo deubiquitination assay, HEK293T cells were harvested 48 h after transfection with the indicated plasmids. For denaturing, lysates were heated at 95 °C for 5 min in the presence of 1% SDS, followed by tenfold dilution with lysis buffer (to 0.1% SDS) and sonication, as described previously^24^. The cell lysates were incubated with S-protein or anti-MYC beads for 2 h, and then the beads were washed with lysis buffer three times and subjected to immunoblotting analysis. For the in vitro deubiquitination assay, HEK293T cells were transfected with HA-ubiquitin and SFB-YAP plasmids; 48 h after transfection, SFB-YAP was pulled down by S-protein beads and incubated with bacterially purified His-OTUD1 protein (wild-type or C320S) at 37 °C for 3 h in deubiquitination buffer (50 mM Tris-HCl, pH 8.0, 50 mM NaCl, 10 mM DTT, 1 mM EDTA, 5% glycerol). After the reaction, the beads were washed with deubiquitination buffer, and the bound proteins were eluted by boiling in 1 × Laemmli buffer and subjected to western blot analysis with the indicated antibodies.

### In vitro cell growth assay

The indicated HMLE, MDA-MB-231, or BT549 cells were seeded in 96-well plates (2000 cells per well). Cells were fixed at indicated times and stained with crystal violet (0.05% w/v in formalin). The dye from stained cells was dissolved in 10% acetic acid and the absorbance was measured at 570 nm.

### Soft agar assay

In all, 2.5 × 10^4^ HMLE cells were mixed with 0.3% agar in MEGM and seeded in 6-well plates with a bottom layer of 0.6% agar in DMEM. After 3 weeks, colonies were photographed and counted.

### In vivo tumorigenesis study

Animal experiments were performed in accordance with a protocol approved by the Institutional Animal Care and Use Committee of MD Anderson Cancer Center. Mice were randomly assigned to different groups. When used in a power calculation, our sample size predetermination experiments indicated that 8–10 mice per group can identify the expected effect on tumor growth (*P* < 0.05) with 80% power. In all, 4.5 × 10^6^ tumor cells in 100 μl of growth medium (mixed with Matrigel at a 1:1 ratio) were injected into the mammary fat pads of six-week-old female NSG mice. Tumor size was measured once a week using a caliper, and tumor volume was calculated using the standard formula 0.5 × *L* × *W*^2^, where *L* is the longest diameter and *W* is the shortest diameter. Mice were euthanized when they met the institutional euthanasia criteria for tumor size or overall health condition. The tumors were removed, photographed, and weighed. The investigators were not blinded to the group allocation during experiments and outcome assessment.

### Statistics and reproducibility

Except the animal study, each experiment was repeated three times or more. Unless otherwise noted, data are presented as mean ± s.e.m., and Student’s *t*-test (unpaired, two-tailed) was used to compare two groups of independent samples. The data analyzed by the *t*-test meet normal distribution; we used an *F*-test to compare variances, and the variances are not significantly different. Therefore, when using an unpaired *t*-test, we assumed equal variance, and no data points were excluded from the analysis. Statistical significance of YAP subcellular localization (%) was determined by a chi-square test. *P* < 0.05 was considered statistically significant. *P < 0.05; **P < 0.01; ***P < 0.001.

### Data availability

All source data are available from the corresponding author on reasonable request.

## Electronic supplementary material

Supplementary Information
