# Supplementary material for: SKP2- and OTUD1-regulated non-proteolytic ubiquitination of YAP promotes YAP nuclear localization and activity
Source: Nat Commun. 2018 Jun 11;9:2269. doi: 10.1038/s41467-018-04620-y (PMC5995870; doi:10.1038/s41467-018-04620-y)
Supplement: Supplementary file 1 — Supplementary Information [file 41467_2018_4620_MOESM1_ESM.pdf]

Supplementary Information

Yao et al.

SKP2- and OTUD1-regulated non-proteolytic ubiquitination of YAP promotes YAP nuclear localization and activity

Supplementary Figure 1

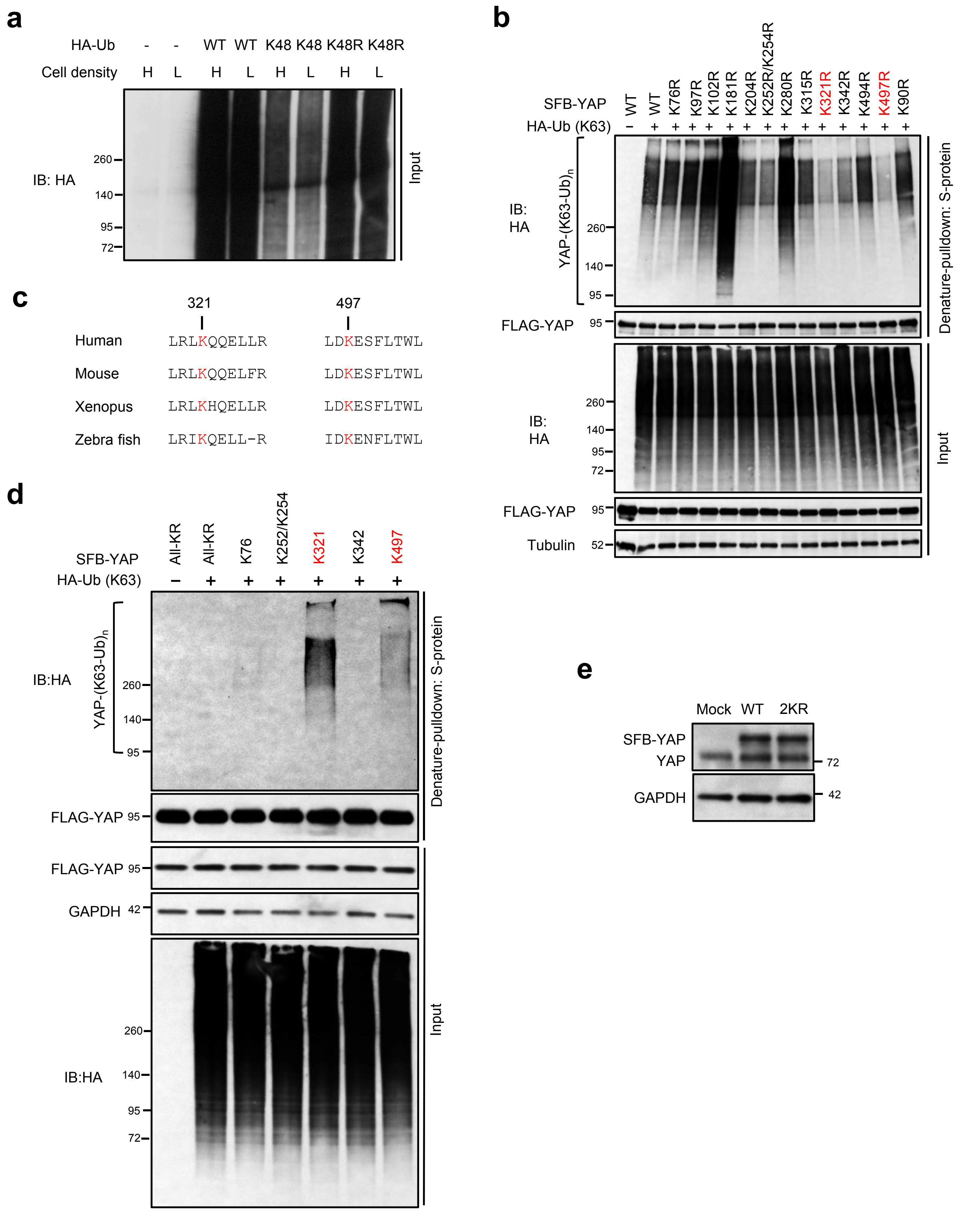

Supplementary Figure 1. K321 and K497 of YAP are the critical sites for its K63-linked ubiquitination. (**a**) Immunoblotting of HA-ubiquitin in the input used in **Fig. 1a**. (**b**) HEK293T cells were transfected with SFB-YAP (wild-type or various KR mutants) and the K63-specific mutant of HA-ubiquitin and then subjected to a pulldown assay with S-protein beads and immunoblotting with antibodies against HA and FLAG. (**c**) Two K63-linked ubiquitination sites (K321 and K497) on human YAP and alignment with conserved sites on mouse, Xenopus and zebra fish Yap. (**d**) The SFB-YAP mutant (all-KR, K76-specific, K252/K254-specific, K321-specific, K342-specific or K497-specific) was co-transfected with or without the K63-specific mutant of HA-ubiquitin into 293T cells, followed by pulldown with S-protein beads and immunoblotting with antibodies against HA and FLAG. (**e**) Immunoblotting of YAP and GAPDH in the samples used in **Fig. 1f, g**.

Supplementary Figure 2

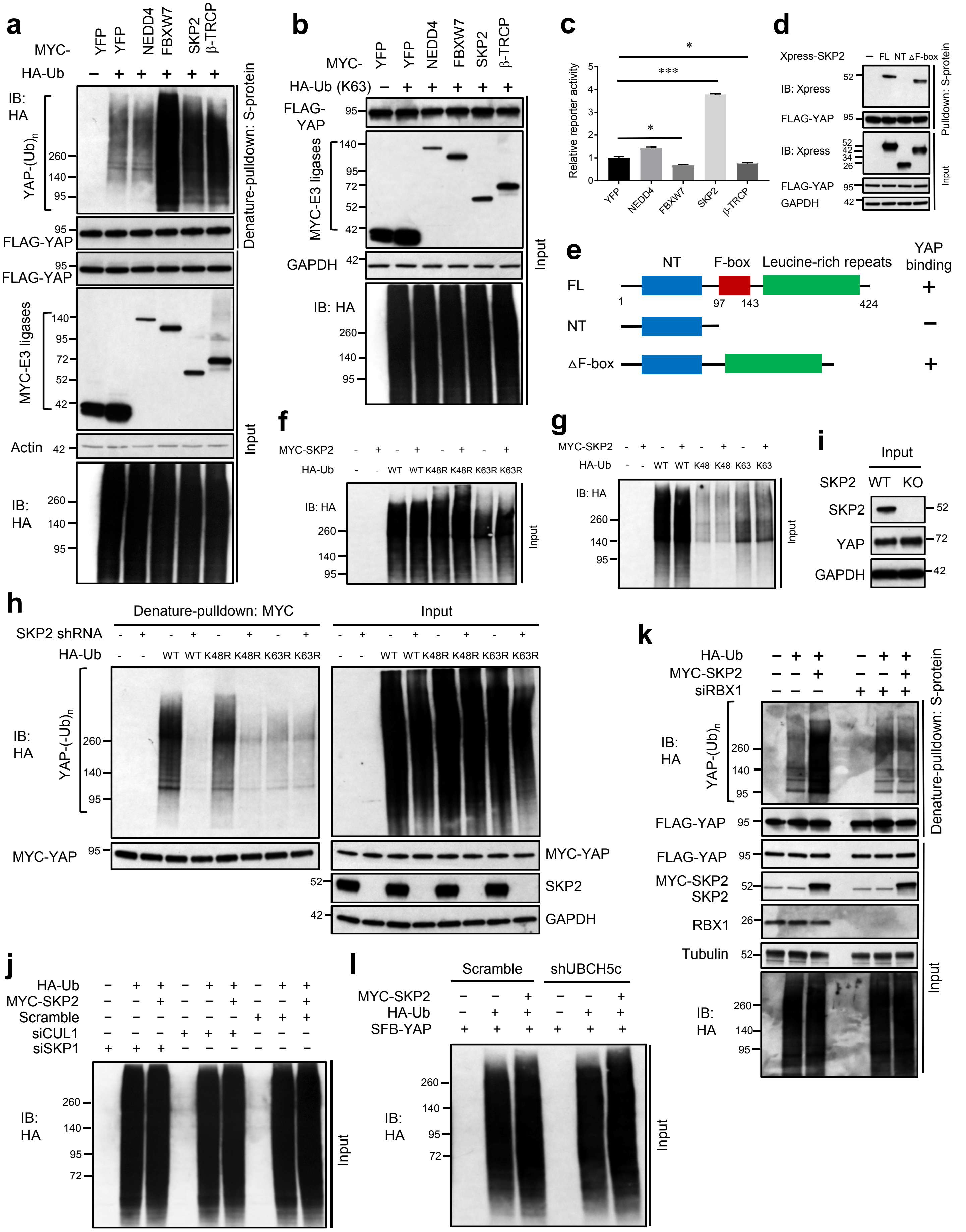

Supplementary Figure 2. SKP2 induces YAP polyubiquitination. (**a**) The HEK293T SFB-YAP stable cell line was co-transfected with HA-ubiquitin and the indicated E3 ligase and then subjected to pulldown with S-protein beads and immunoblotting with antibodies against HA and FLAG. (**b**) Immunoblotting of FLAG-YAP, MYC-E3, GPADH and HA-ubiquitin in the input used in **Fig. 2b**. (**c**) Luciferase activity in HEK293T cells co-transfected with the indicated E3 ligase, an 8× GTIIC luciferase reporter and a TK-Renilla luciferase reporter. Error bars are s.e.m. Statistical significance was determined by a two-tailed, unpaired Student's *t*-test. * $P < 0.05$; ** $P < 0.01$; *** $P < 0.001$. $n = 3$ biological replicates. (**d**) The HEK293T SFB-YAP stable cell line was transfected with Xpress-tagged full-length SKP2 or its deletion mutants, followed by pulldown with S-protein beads and immunoblotting with antibodies against Xpress and FLAG. (**e**) Schematic diagram of full-length (FL) SKP2 and deletion mutants. NT: N-terminal region. (**f**) Immunoblotting of HA-ubiquitin in the input used in **Fig. 2f.** (**g**) Immunoblotting of HA-ubiquitin in the input used in **Fig. 2g**. (**h**) The HEK293T SKP2 shRNA stable cell line was transfected with HA-ubiquitin (wild-type, K48R or K63R) and MYC-YAP, followed by immunoprecipitation with anti-MYC beads and immunoblotting with antibodies against HA and MYC. (**i**) Immunoblotting of SKP2, YAP and GAPDH in the input used in **Fig. 2h**. (**j**) Immunoblotting of HA-ubiquitin in the input used in **Fig. 2i**. (**k**) siRNA targeting *RBX1* was transfected into the HEK293T SFB-YAP stable cell line. 48 hours after siRNA transfection, cells were transfected with HA-ubiquitin and MYC-SKP2 and then subjected to a pulldown assay with S-protein beads and immunoblotting with antibodies against HA and FLAG. (**l**) Immunoblotting of HA-ubiquitin in the input used in **Fig. 2j**.

Supplementary Figure 3

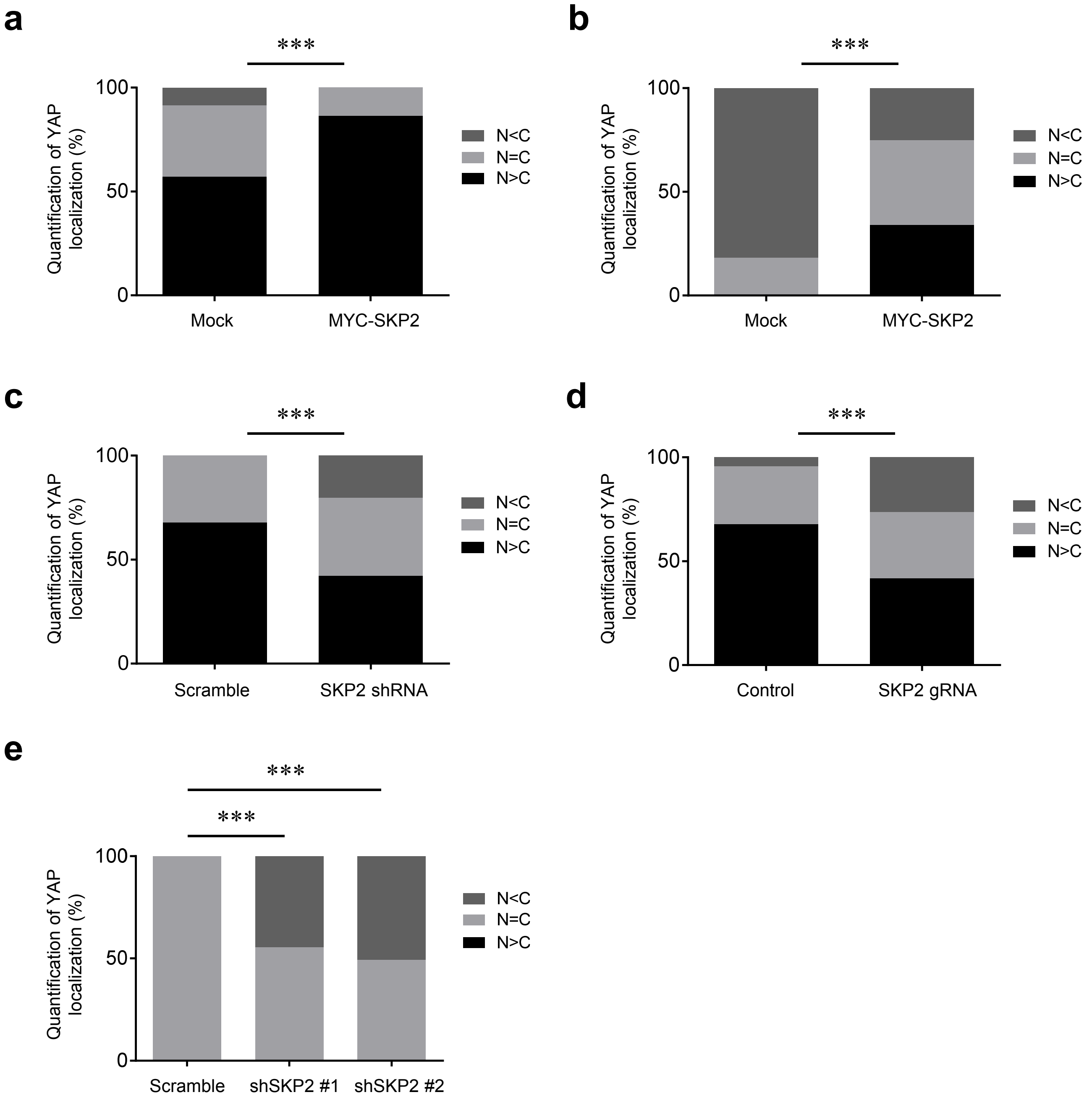

Supplementary Figure 3. SKP2 promotes nuclear localization of YAP. (**a-e**) Data quantification for **Fig. 3a-e**. Statistical significance was determined by a chi-square test. * $P < 0.05$; ** $P < 0.01$; *** $P < 0.001$.

Supplementary Figure 4

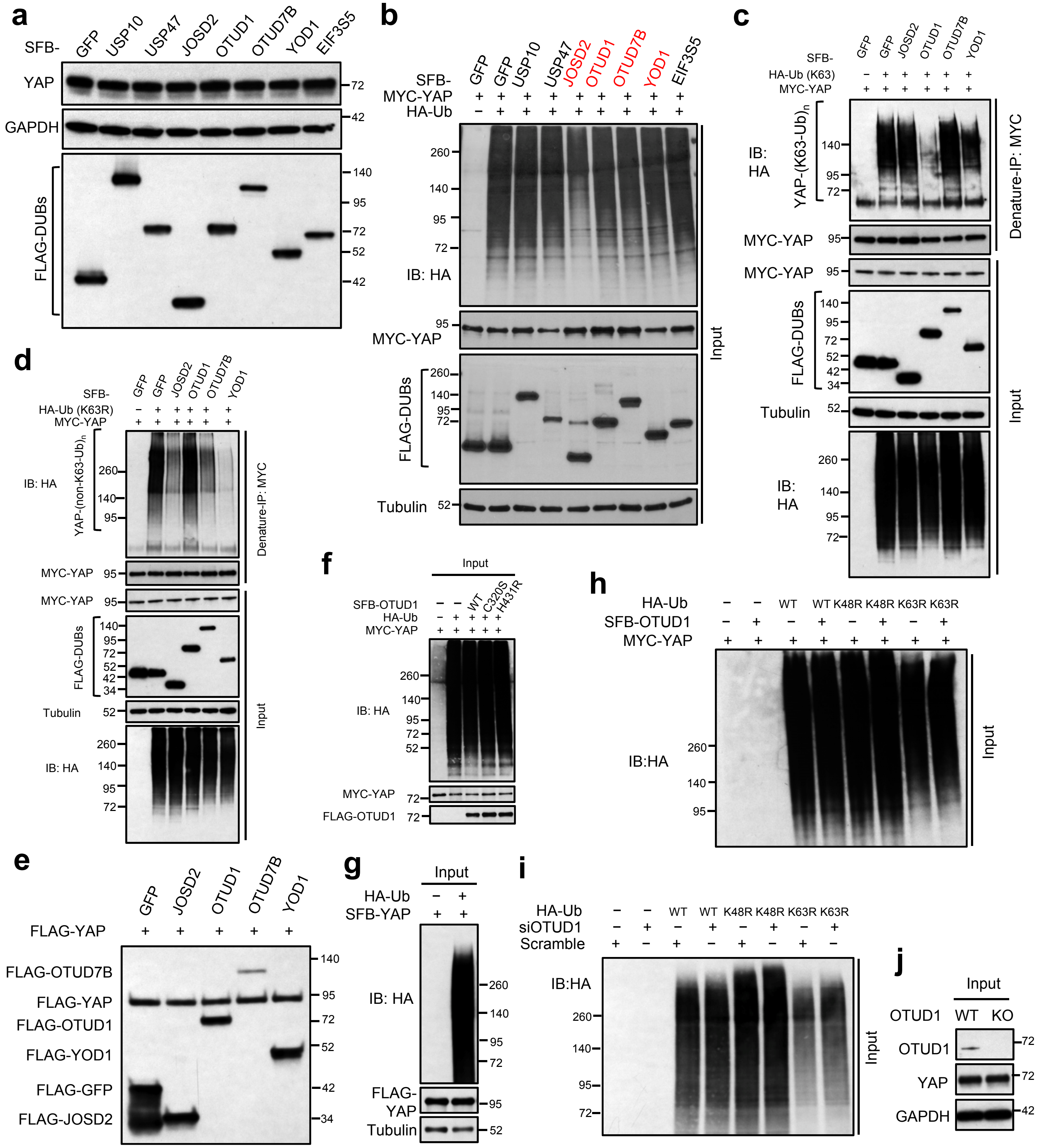

Supplementary Figure 4. OTUD1 is a K63-linkage specific YAP deubiquitinase. (**a**) HEK293T cells were transfected with SFB-tagged DUBs and immunoblotted with antibodies against YAP, GAPDH and FLAG. (**b**) Immunoblotting of HA-ubiquitin, MYC-YAP, FLAG-DUBs and α-tubulin in the input used in **Fig. 4b**. (**c, d**) HEK293T cells were co-transfected with SFB-DUB, MYC-YAP and the K63-specific (**c**) or K63R (**d**) mutant of HA-ubiquitin, followed by immunoprecipitation with anti-MYC beads and immunoblotting with antibodies against HA and MYC. (**e**) Immunoblotting of FLAG-DUBs in the lysates used in **Fig. 4c**. (**f**) Immunoblotting of HA-ubiquitin, MYC-YAP and FLAG-OTUD1 in the input used in **Fig. 4f**. (**g**) Immunoblotting of HA-ubiquitin, FLAG-YAP and α-tubulin in the input used in **Fig. 4g**. (**h**) Immunoblotting of HA-ubiquitin in the input used in **Fig. 4h**. (**i**) Immunoblotting of HA-ubiquitin in the input used in **Fig. 4i**. (**j**) Immunoblotting of OTUD1, YAP and GAPDH in the input used in **Fig. 4j**.

Supplementary Figure 5

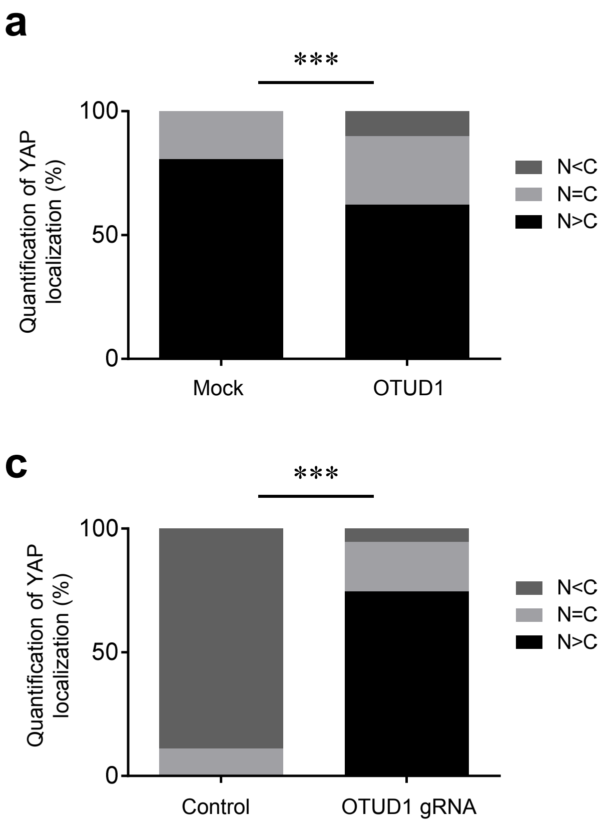

Supplementary Figure 5. OTUD1 promotes cytoplasmic localization of YAP. (**a-c**) Data quantification for **Fig. 5b-d**. Statistical significance was determined by a chi-square test. * $P < 0.05$; ** $P < 0.01$; *** $P < 0.001$.

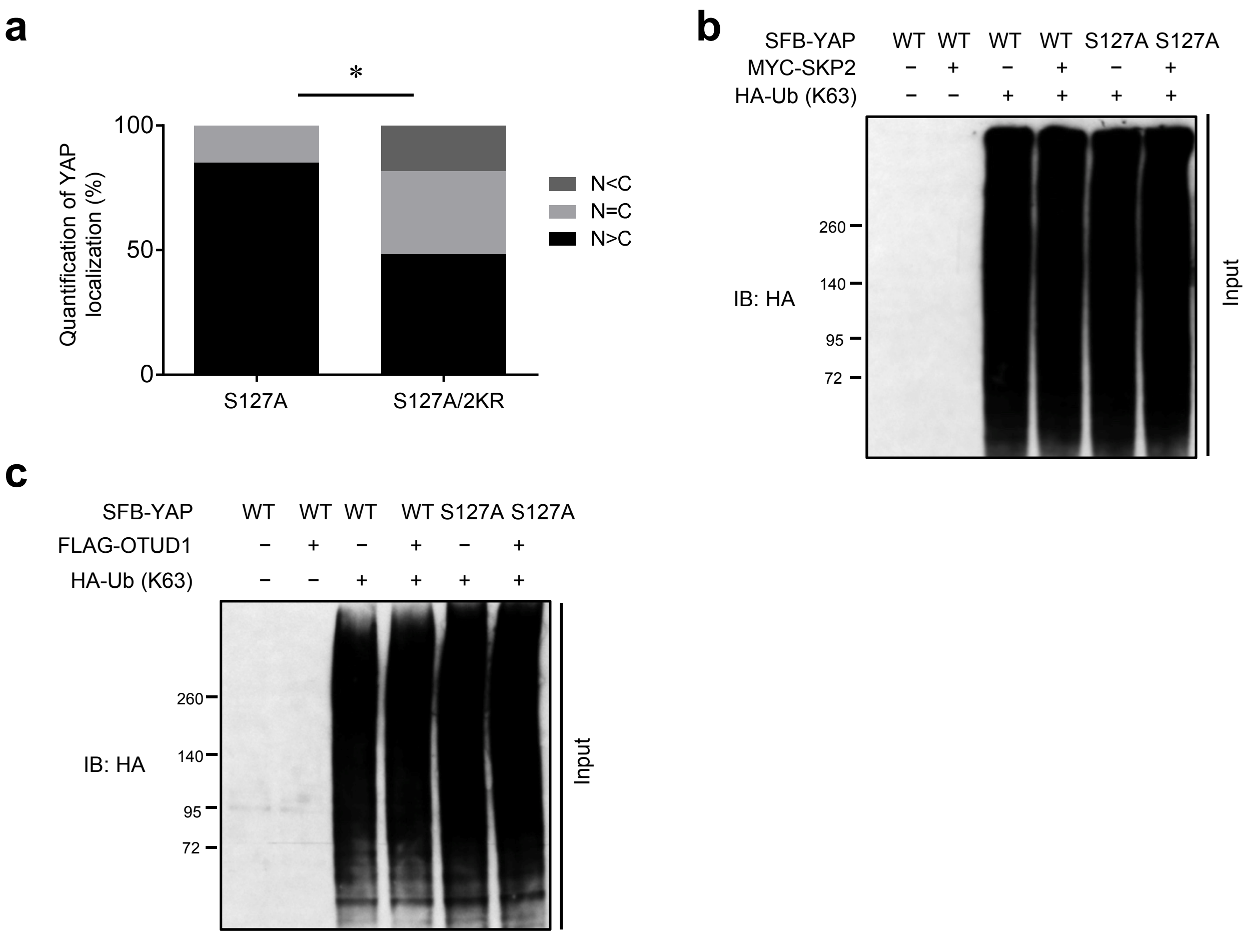

Supplementary Figure 6. K63-linked ubiquitination of YAP is independent of its S127 phosphorylation. (**a**) Data quantification for **Fig. 6b**. Statistical significance was determined by a chi-square test. * *P* < 0.05; ** *P* < 0.01; *** *P* < 0.001. (**b**) Immunoblotting of HA-ubiquitin in the input used in **Fig. 6e**. (**c**) Immunoblotting of HA-ubiquitin in the input used in **Fig. 6f**.

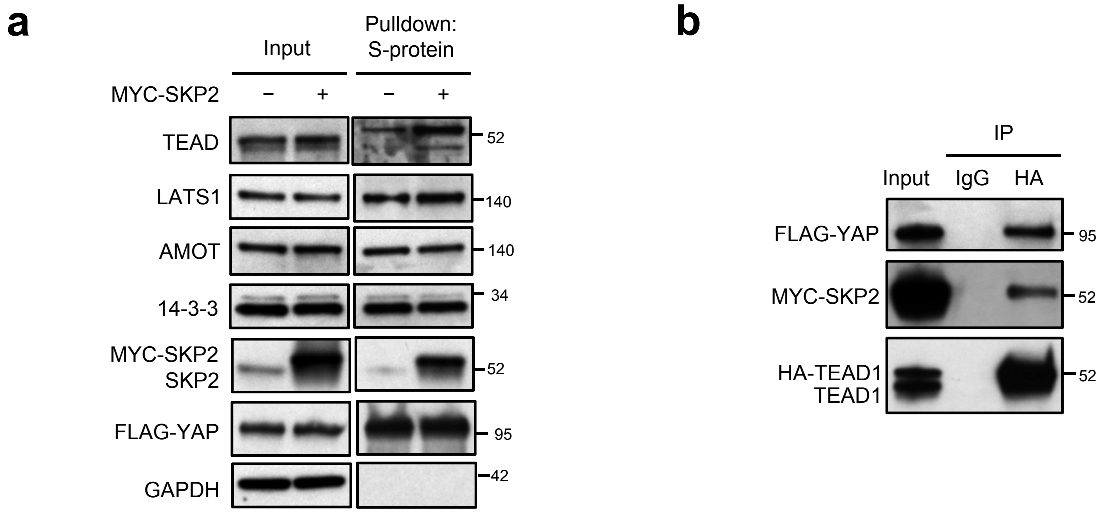

Supplementary Figure 7. SKP2 promotes the interaction between YAP and TEAD. (**a**) BT549 cells were co-transfected with SFB-YAP and MYC-SKP2, followed by pulldown with S-protein beads and immunoblotting with antibodies against YAP-interacting proteins. (**b**) The HEK293A SFB-YAP stable cell line was co-transfected with HA-TEAD1 and MYC-SKP2, followed by pulldown with an HA-specific antibody and immunoblotting with antibodies against FLAG, SKP2 and TEAD1.

Supplementary Figure 8

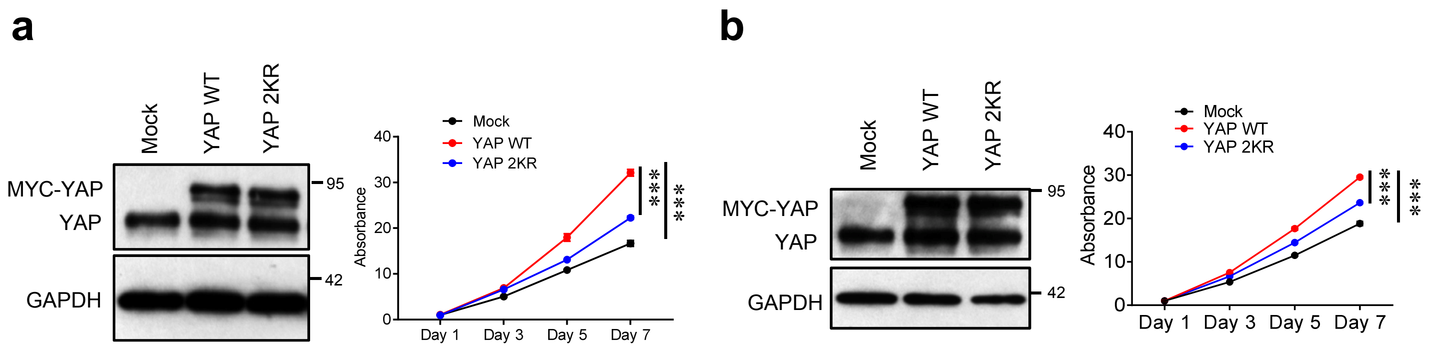

Supplementary Figure 8. K63-linked ubiquitination of YAP activates its growth-promoting function. (**a**) Left panel: immunoblotting of YAP and GAPDH in MDA-MB-231 cells transduced with wild-type (WT) YAP or the K321R/K497R mutant (2KR). Right panel: growth curves. *n* = 5 biological replicates. (**b**) Left panel: immunoblotting of YAP and GAPDH in BT549 cells transduced with wild-type (WT) YAP or the K321R/K497R mutant (2KR). Right panel: growth curves. *n* = 5 biological replicates. Error bars in (**a**) and (**b**) are s.e.m. Statistical significance was determined by a two-tailed, unpaired Student's *t*-test. * $P < 0.05$; ** $P < 0.01$; *** $P < 0.001$.

Supplementary Figure 9

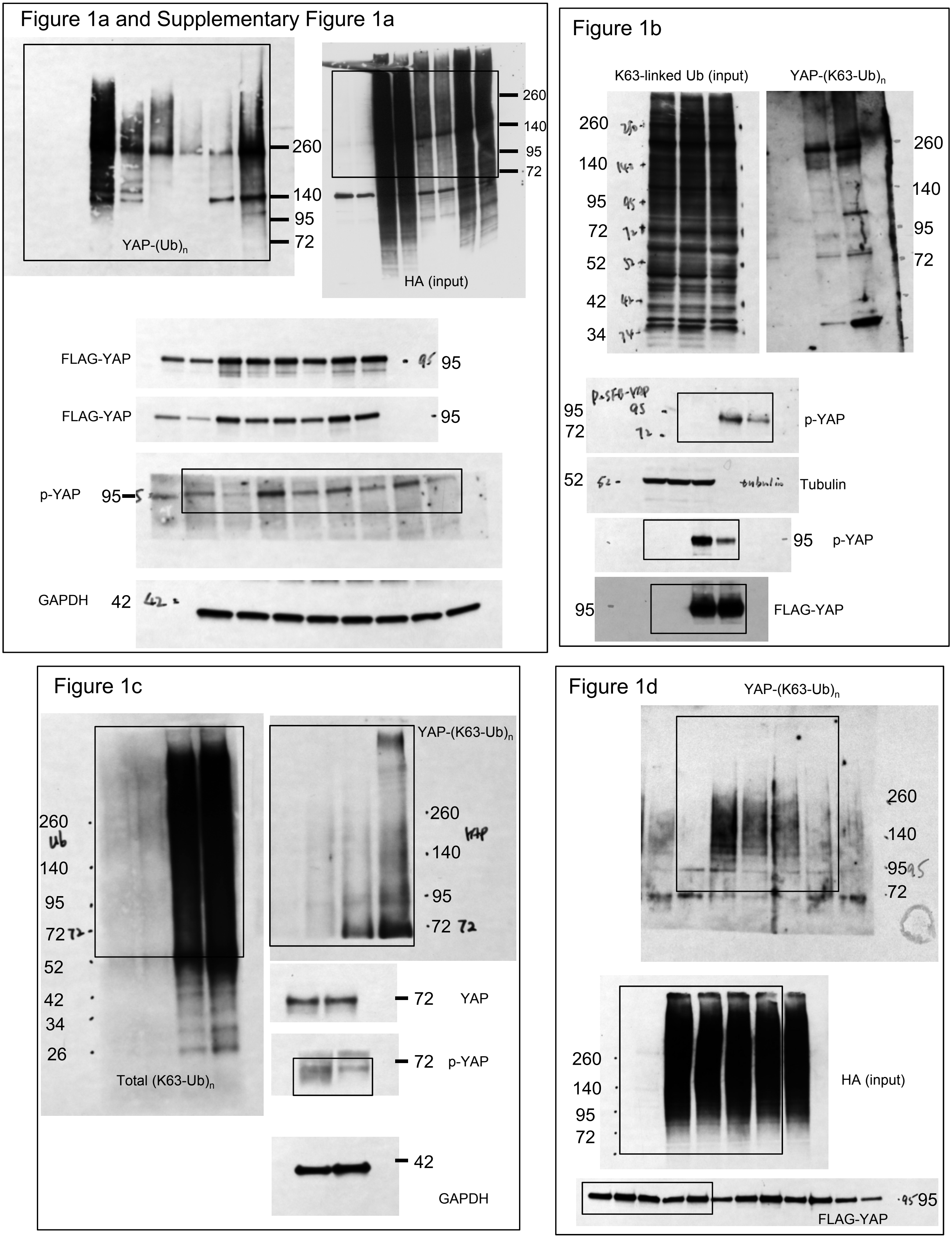

Figure 1e

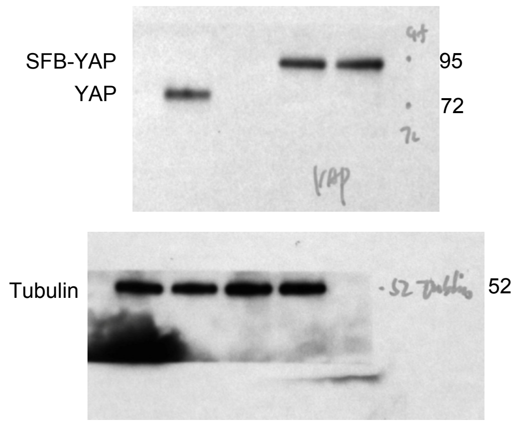

Figure 2a

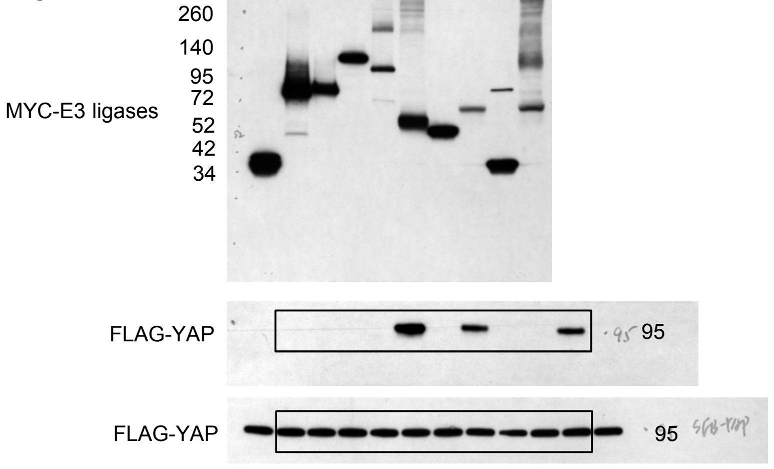

Figure 2b and Supplementary Figure 2b

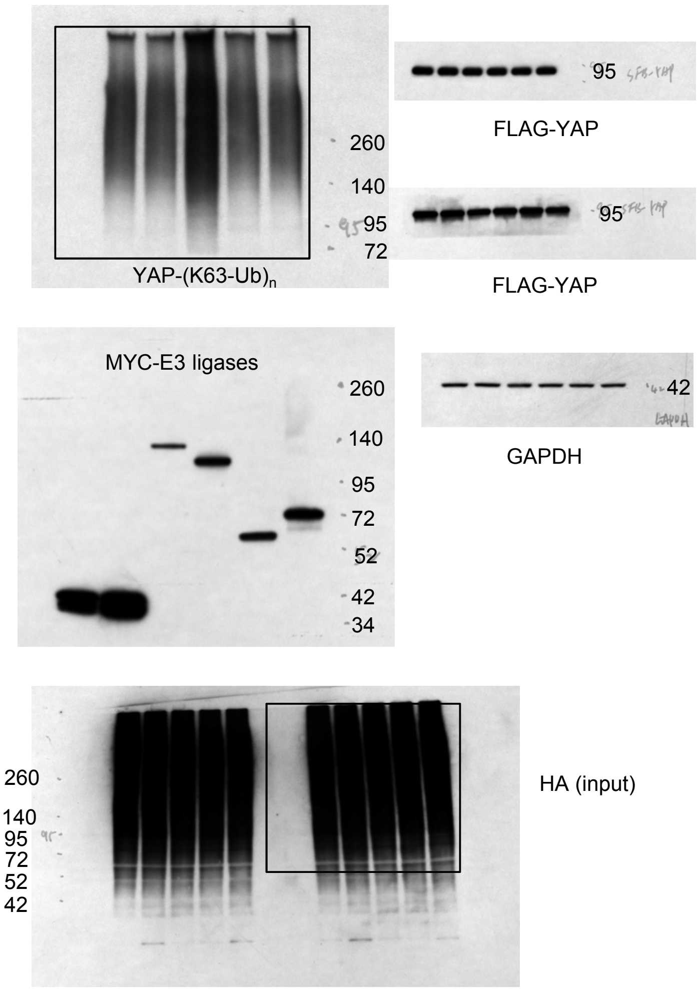

Figure 2c

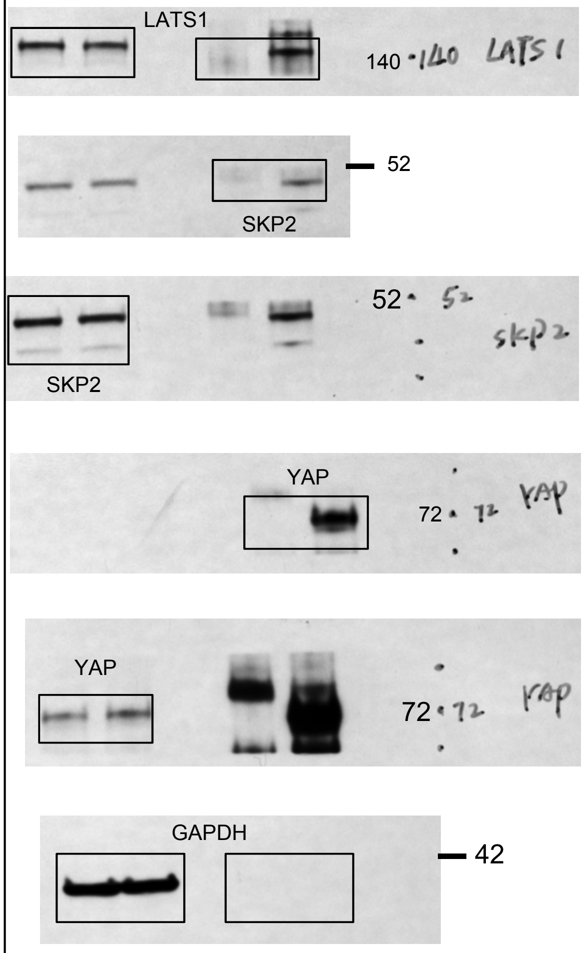

Figure 2d

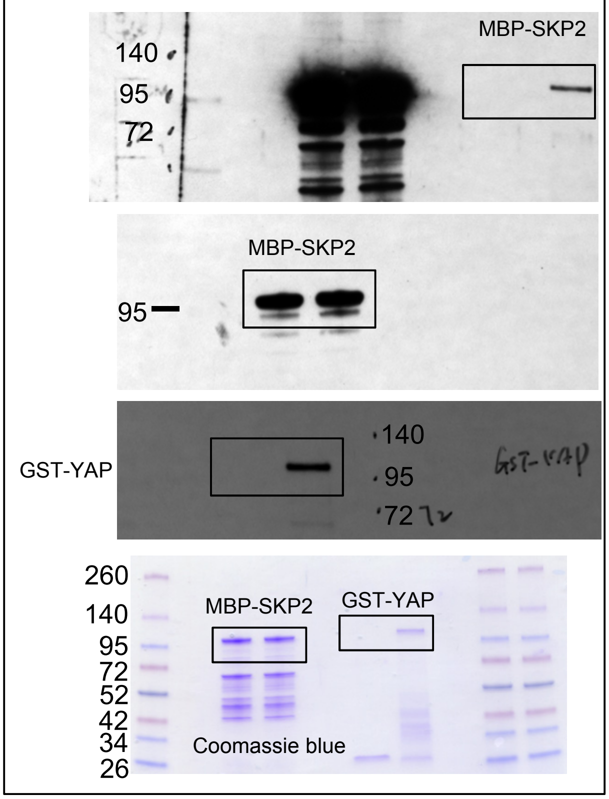

Figure 2e

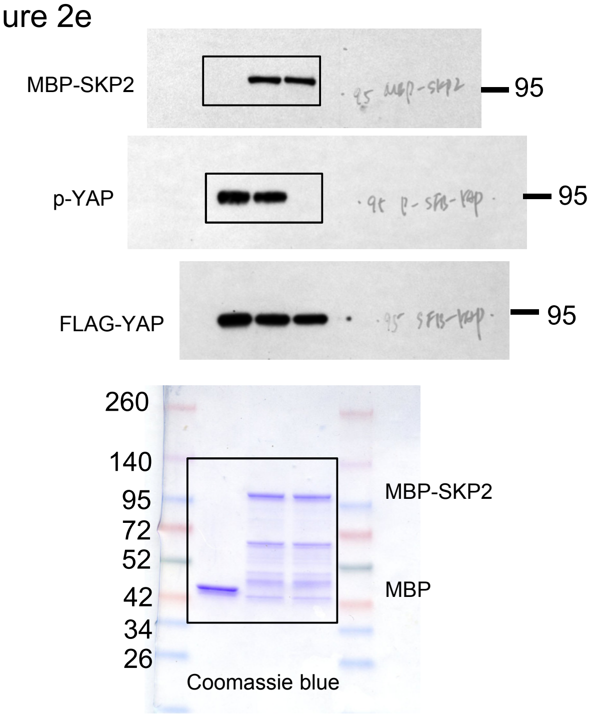

Figure 2f and Supplementary Figure 2f

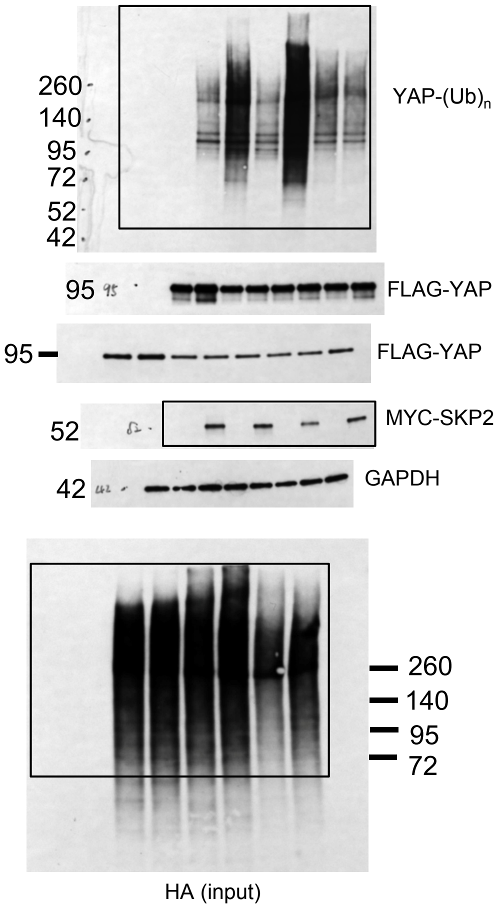

Figure 2g and Supplementary Figure 2g

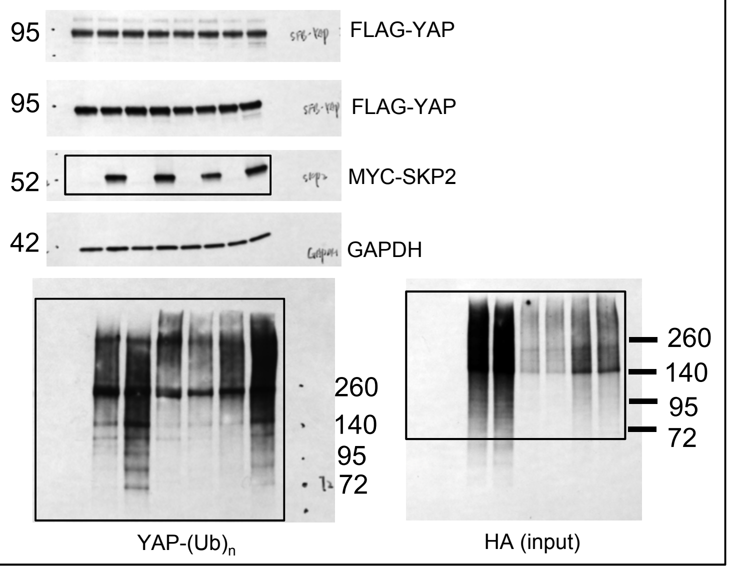

Figure 2h and Supplementary Figure 2i

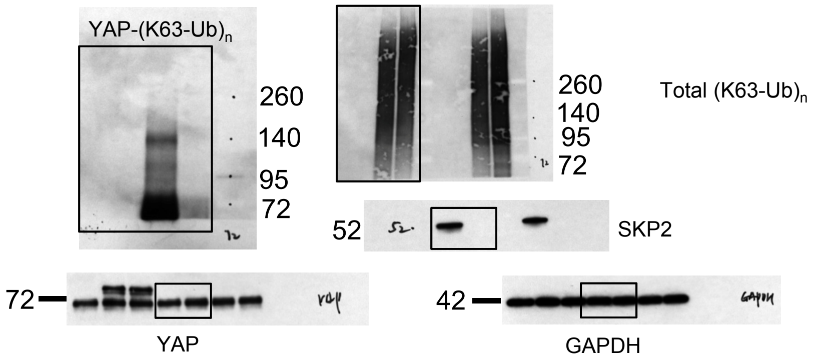

Figure 2i and Supplementary Figure 2j

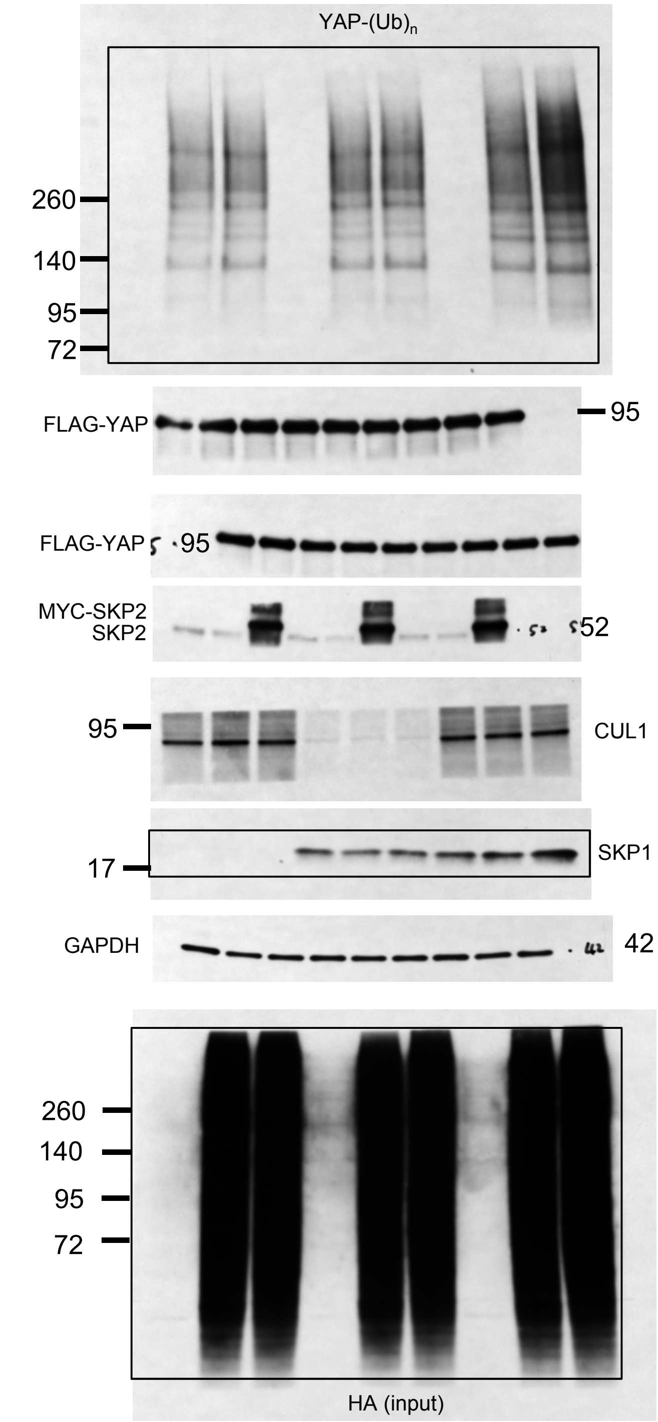

Figure 2j and Supplementary Figure 2l

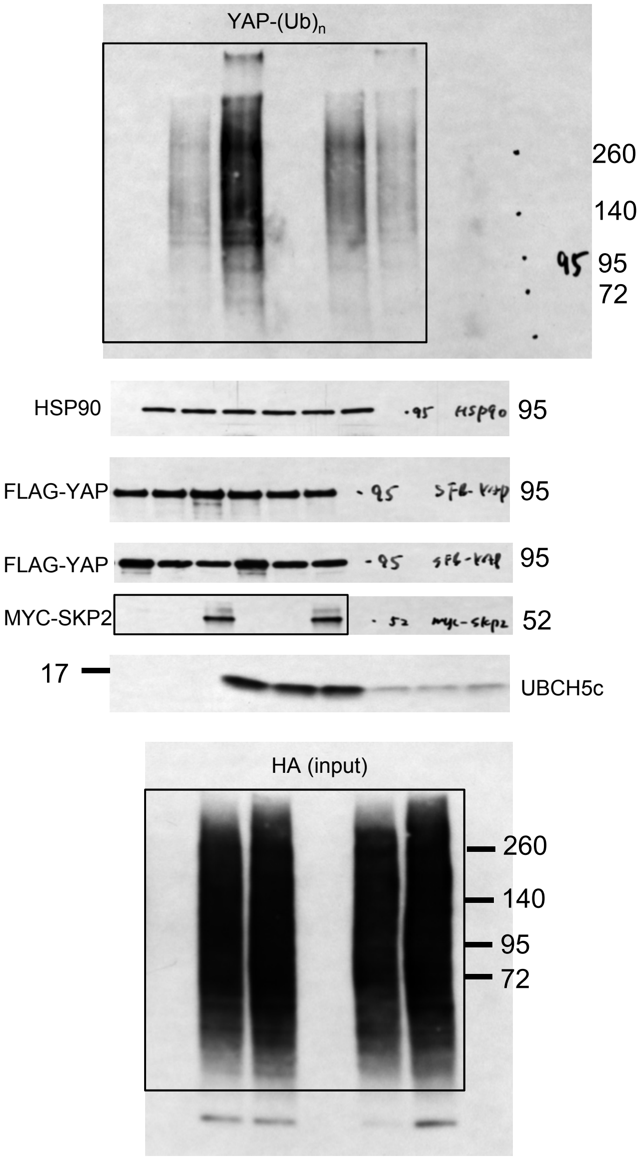

Figure 2k

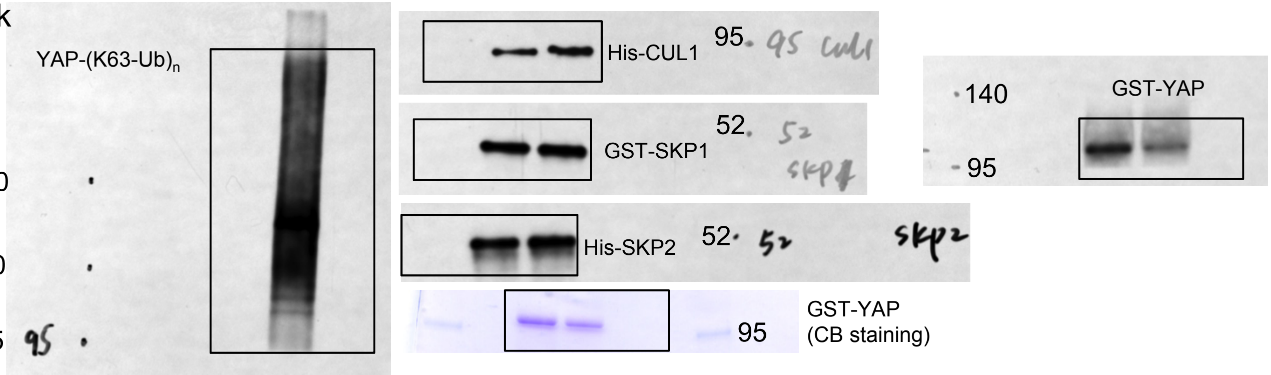

Figure 3i

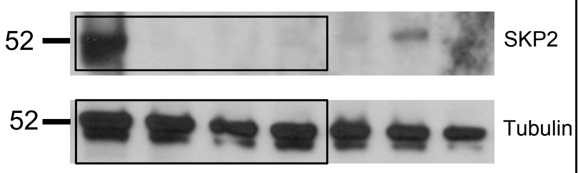

Figure 4a

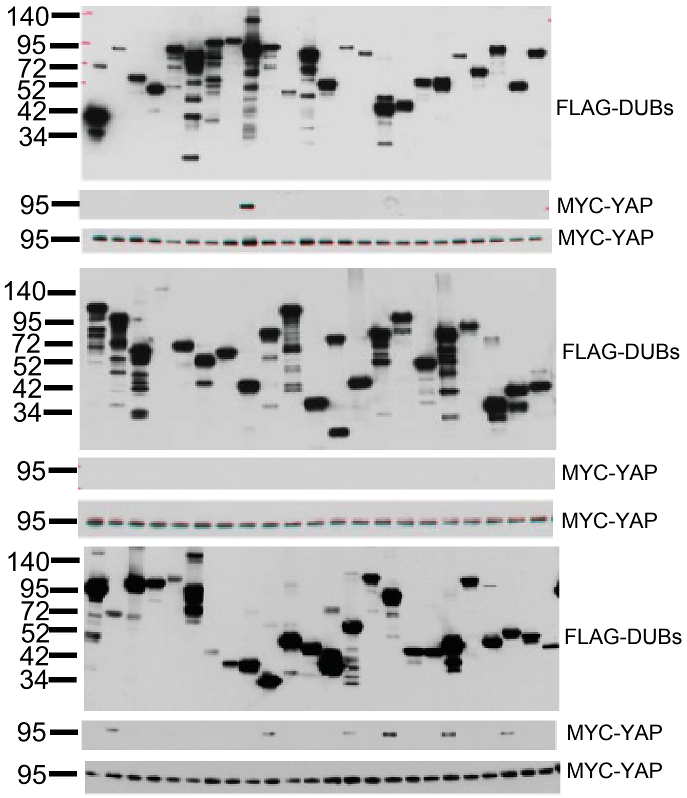

Figure 4b and Supplementary Figure 4b

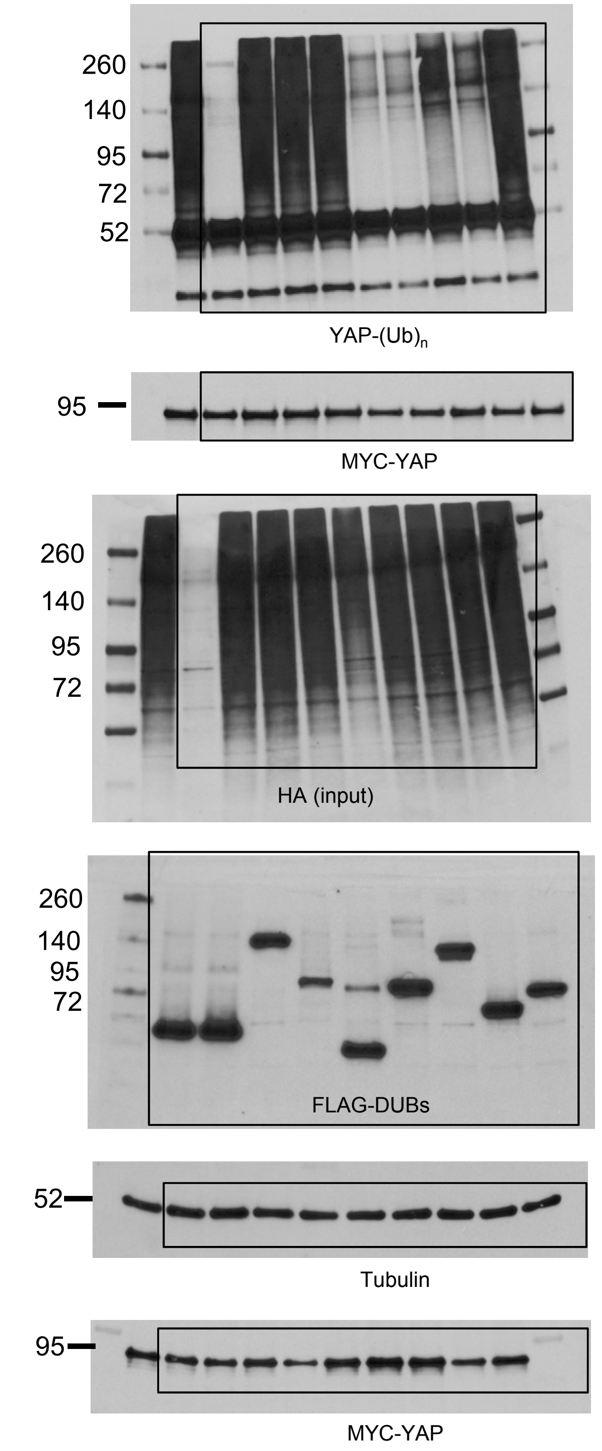

Figure 4d

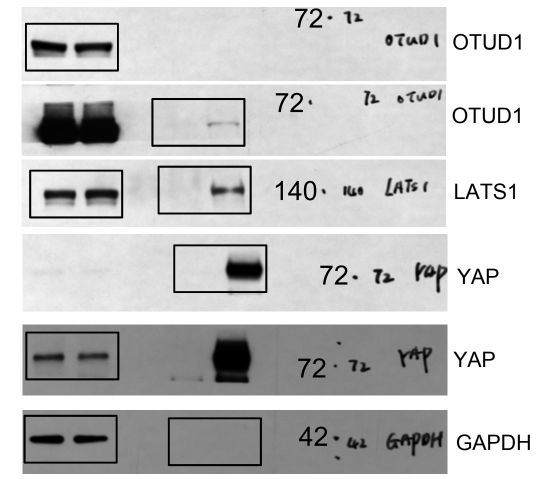

Figure 4e

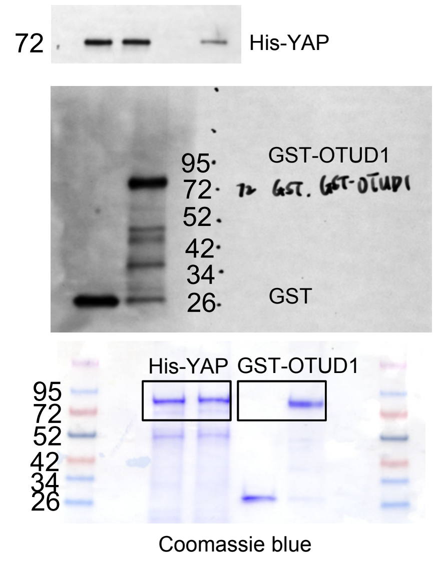

Figure 4f and Supplementary Figure 4f

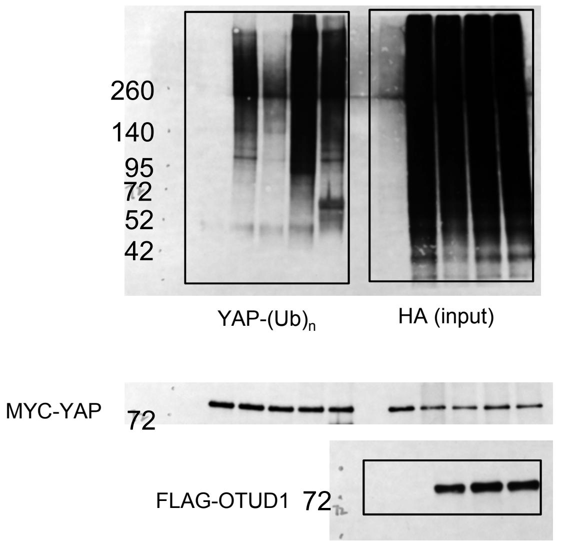

Figure 4g and Supplementary Figure 4g

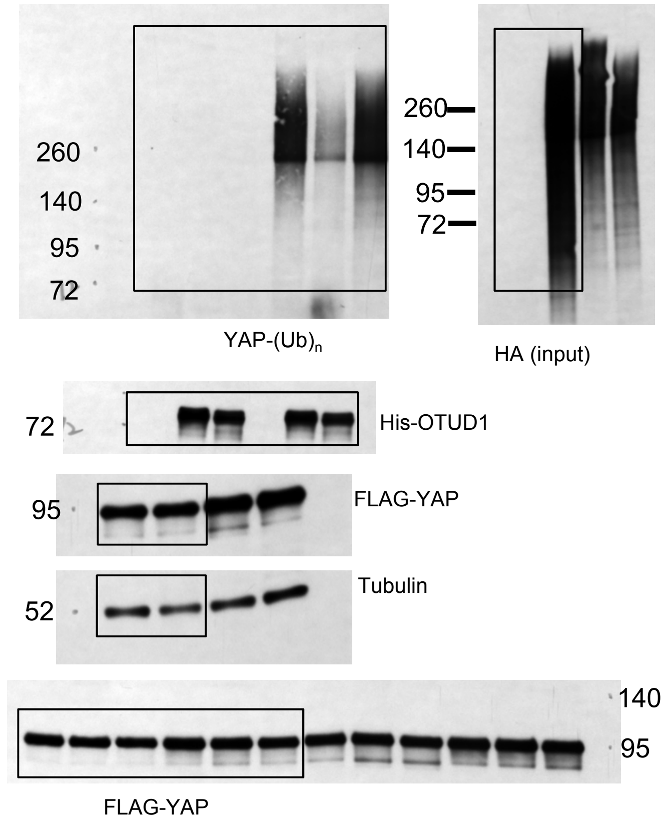

Figure 4h and Supplementary Figure 4h

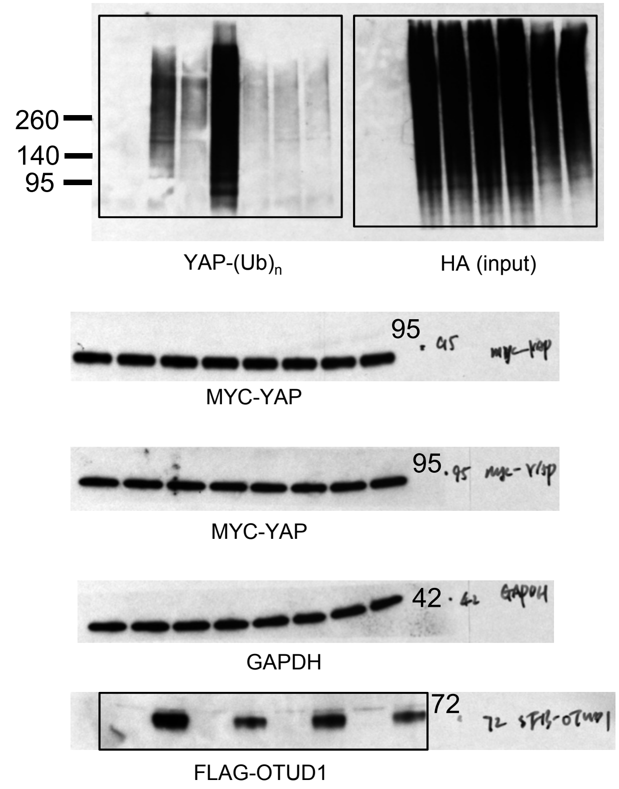

Figure 4i and Supplementary Figure 4i

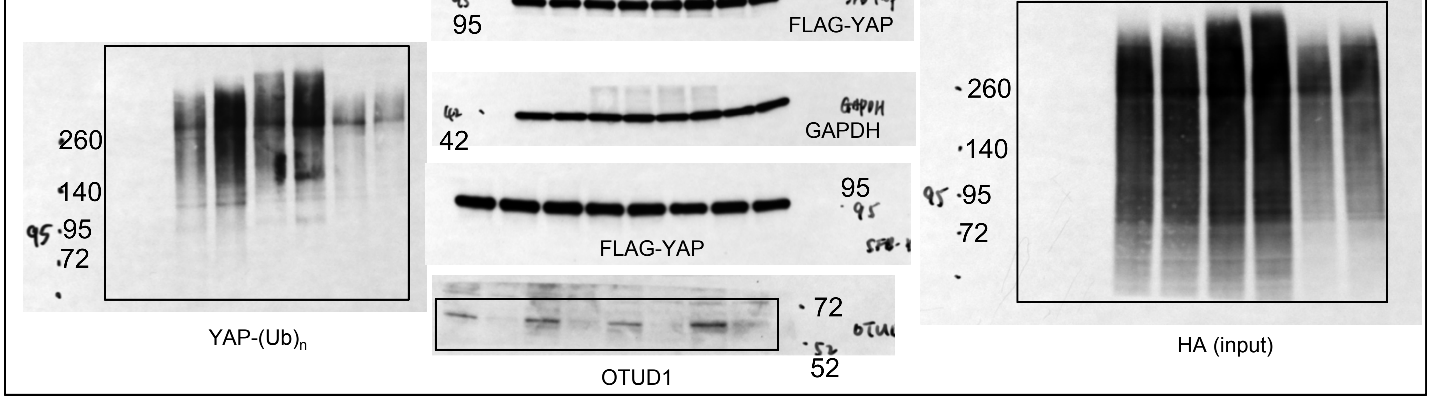

Figure 4j and Supplementary Figure 4j

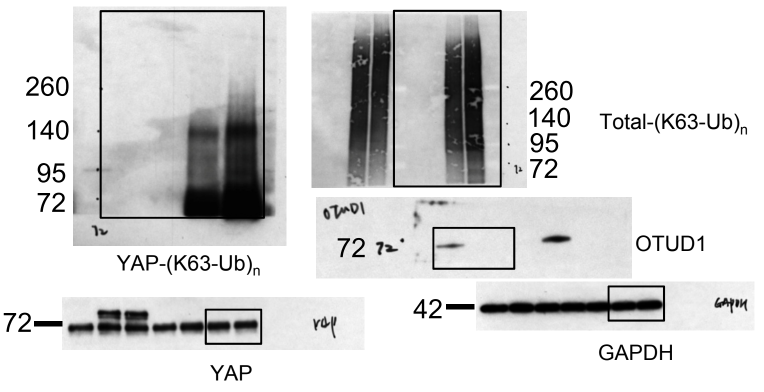

Figure 5a

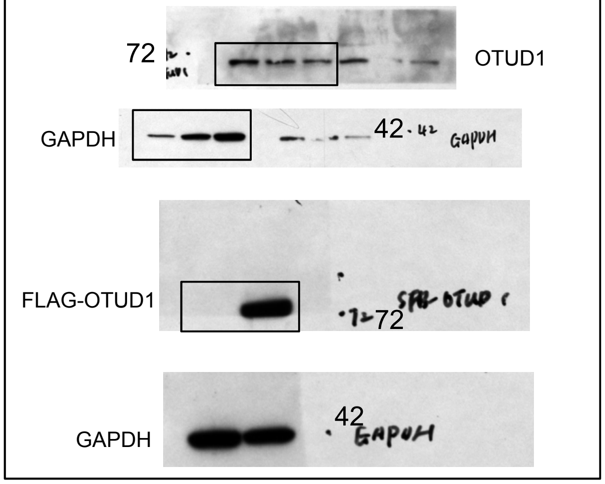

Figure 5g

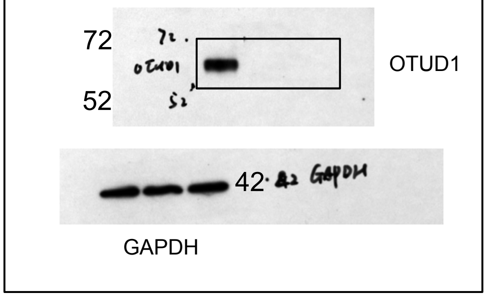

Figure 6a

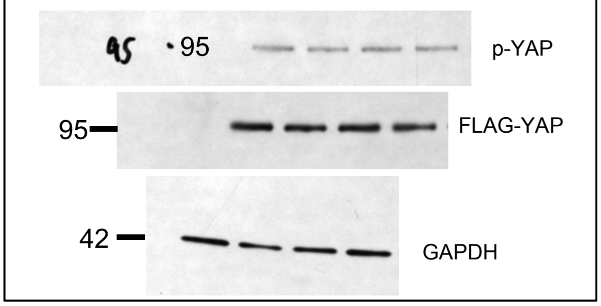

Figure 6c

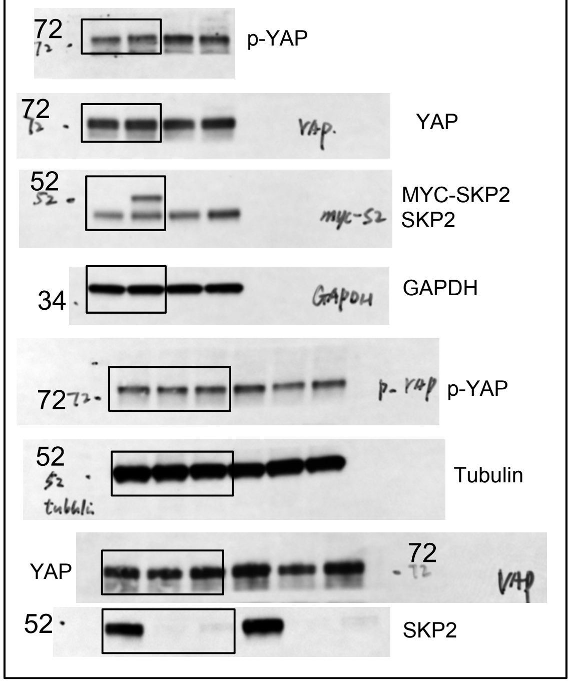

Figure 6d

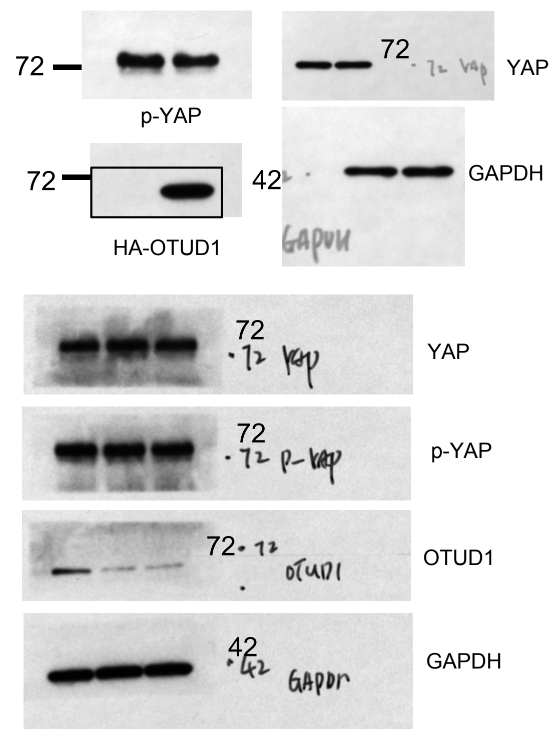

Figure 6e and Supplementary Figure 6b

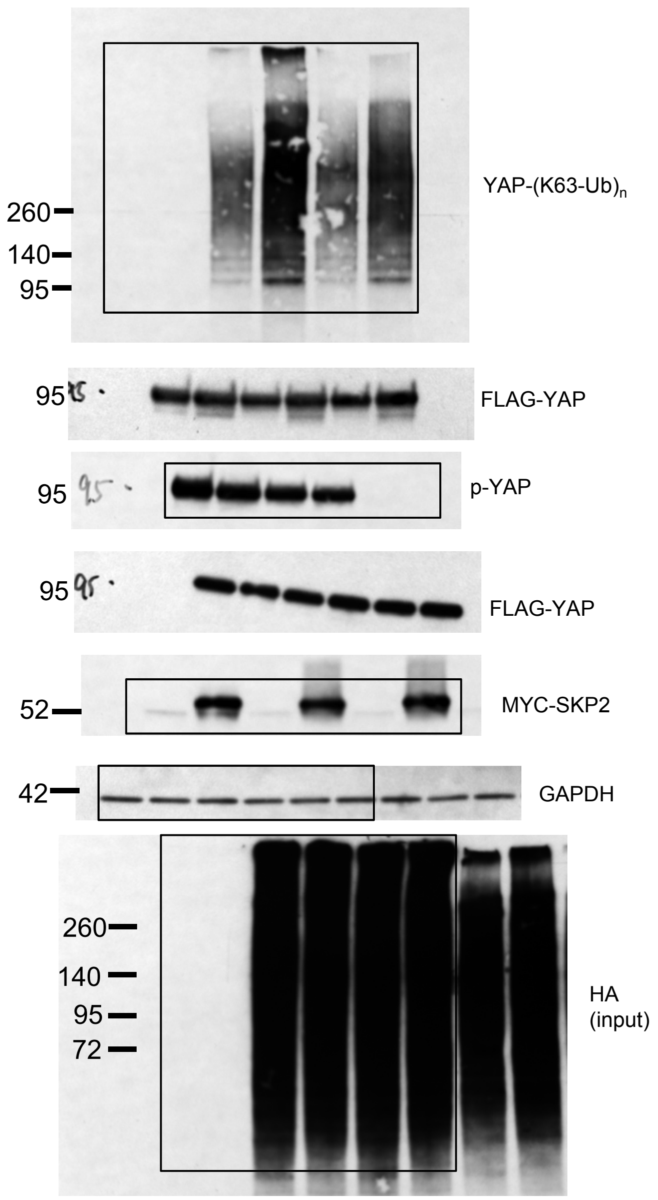

Figure 6f and Supplementary Figure 6c

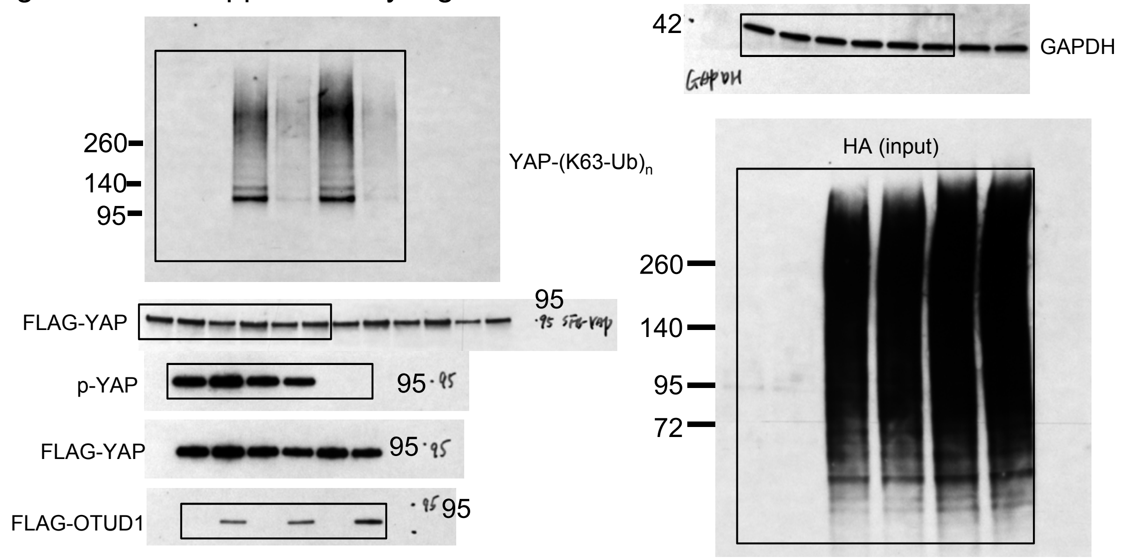

Figure 7a

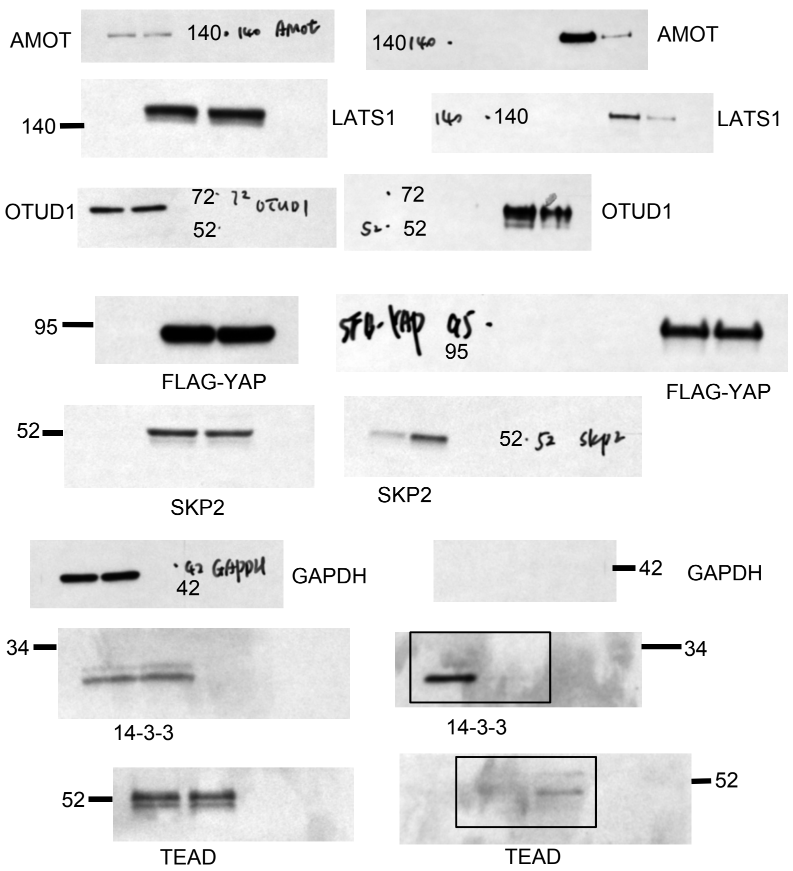

Figure 7b

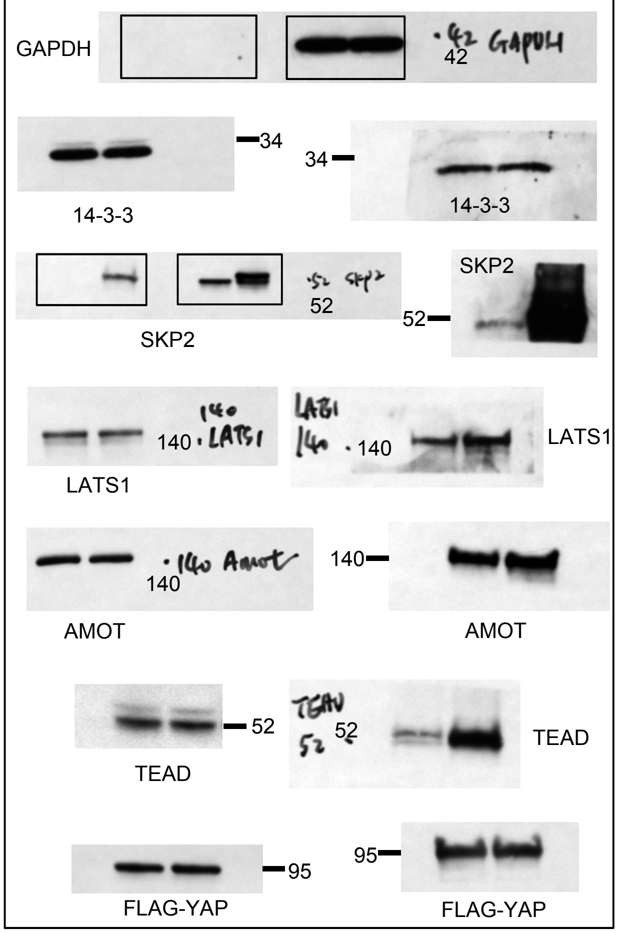

Figure 7c

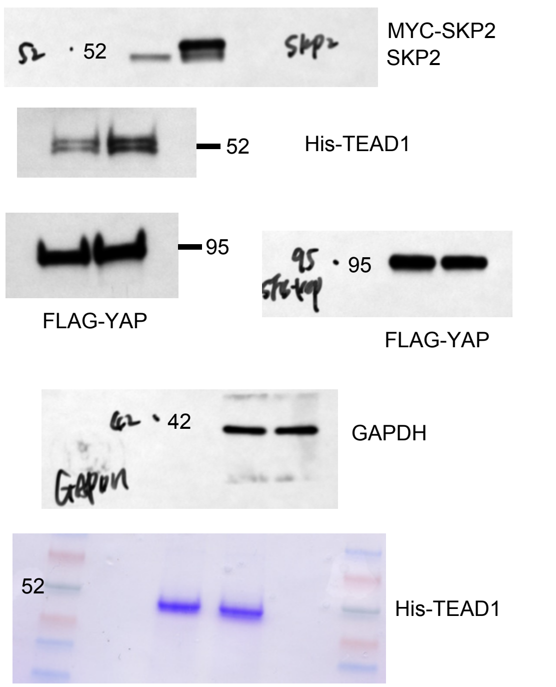

Figure 7d

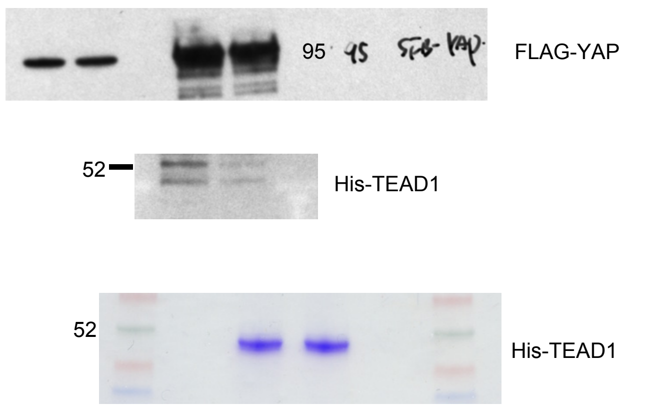

Figure 7e

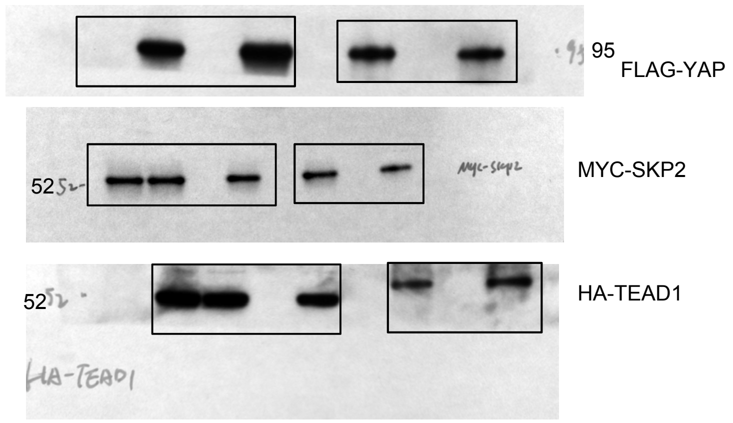

Figure 8a

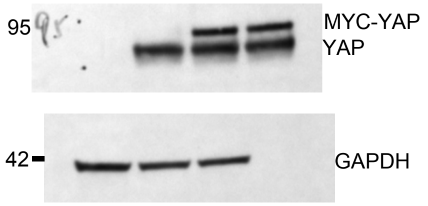

Figure 8f

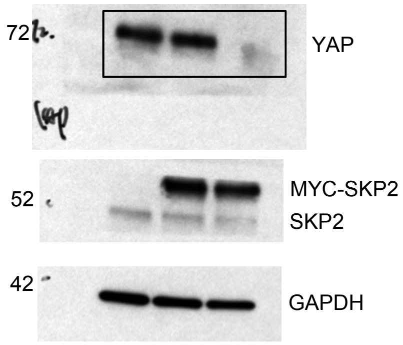

Figure 8g

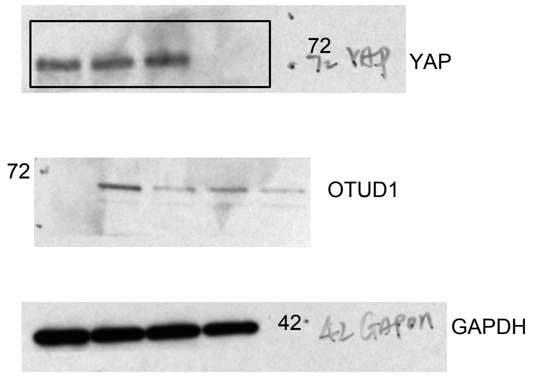

Supplementary Figure 1b

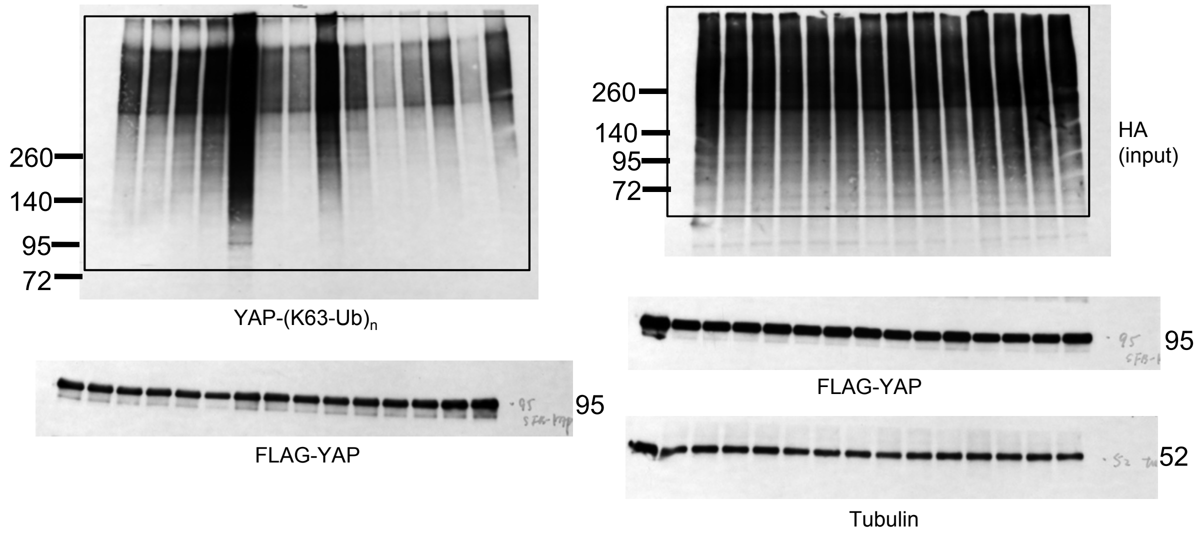

Supplementary Figure 1d

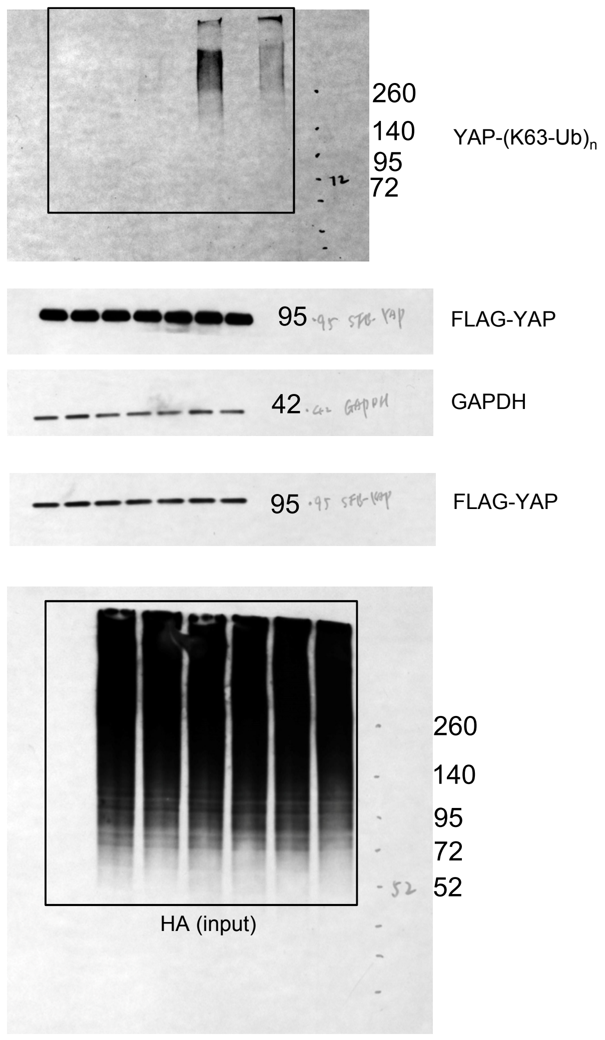

Supplementary Figure 1e

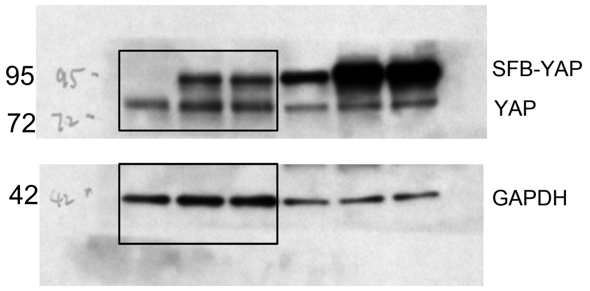

Supplementary Figure 2a

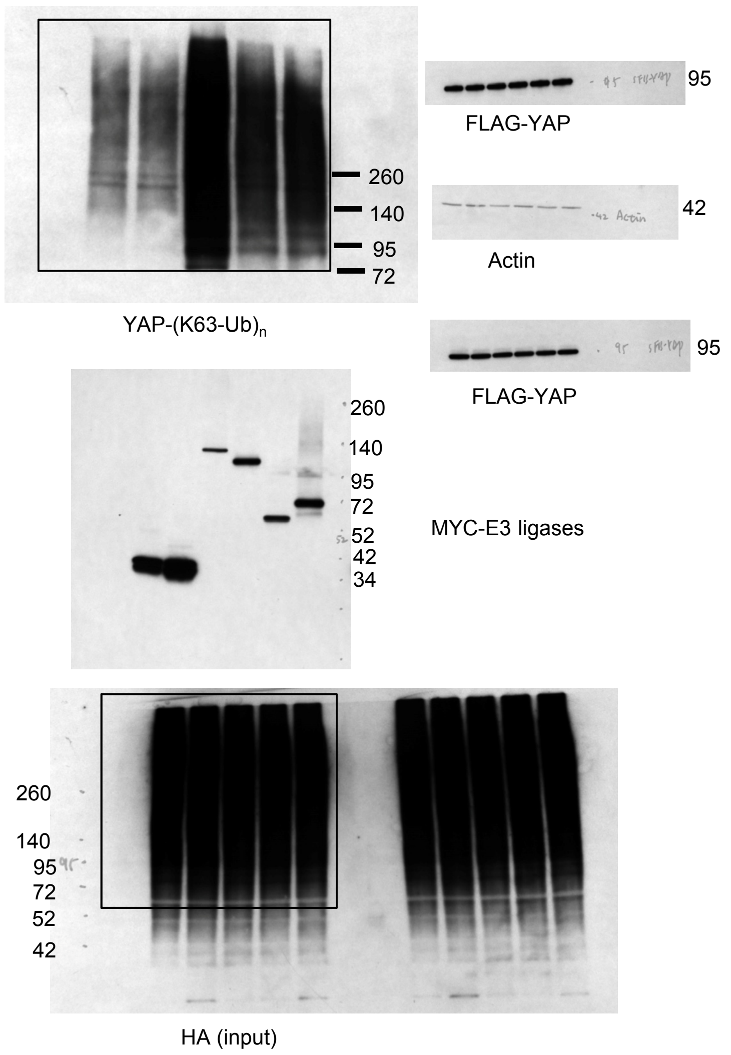

Supplementary Figure 2d

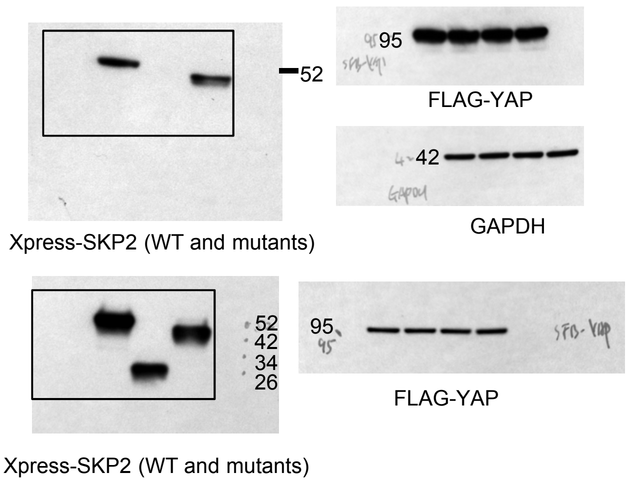

Supplementary Figure 2h

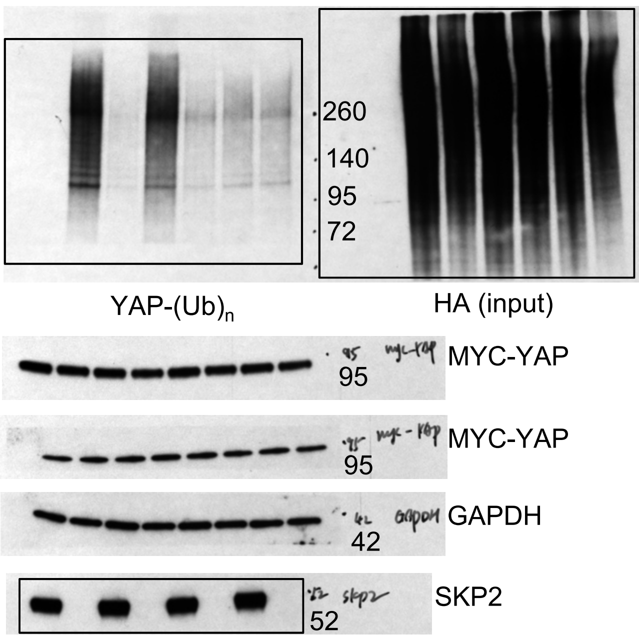

Supplementary Figure 2k

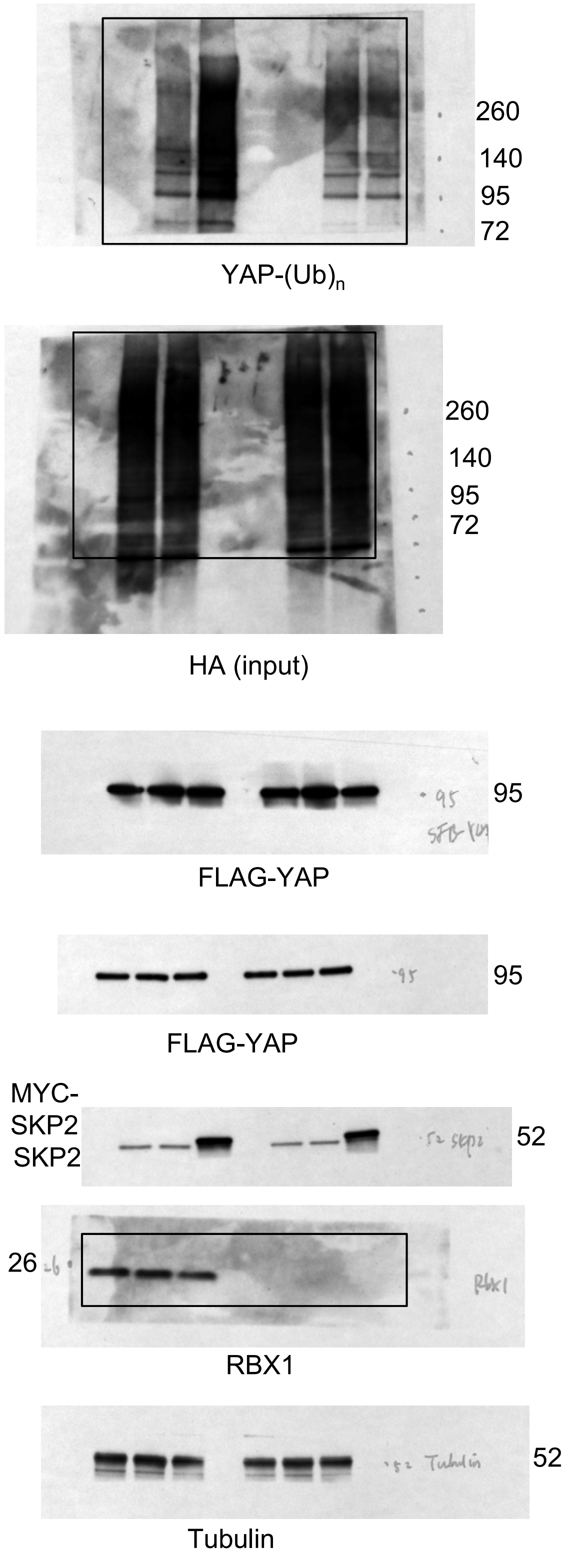

Supplementary Figure 4a

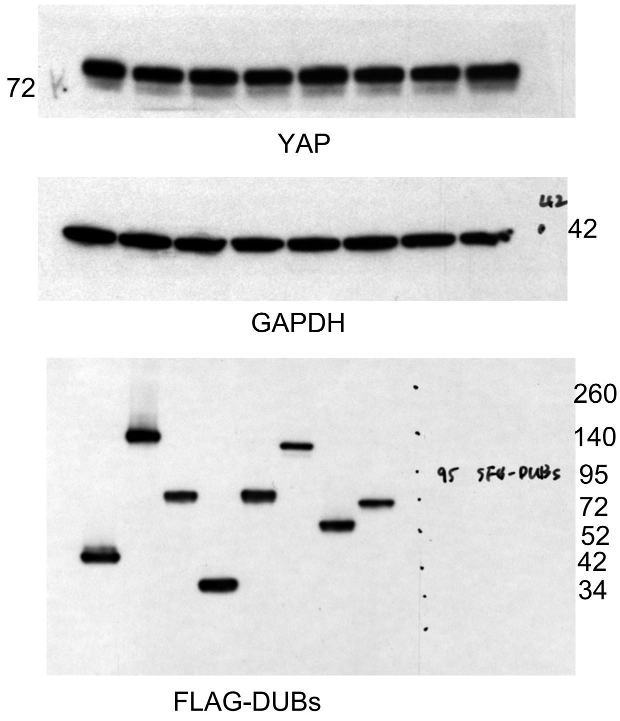

Supplementary Figure 4c

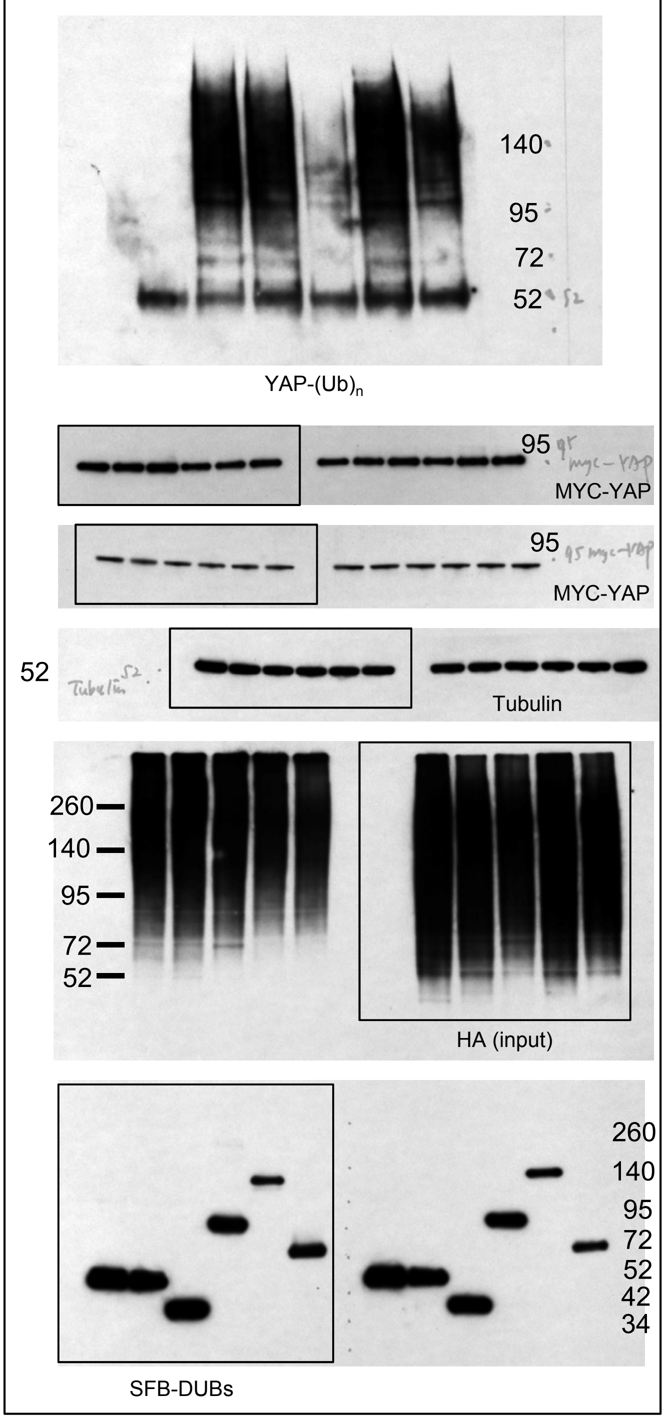

Supplementary Figure 4d

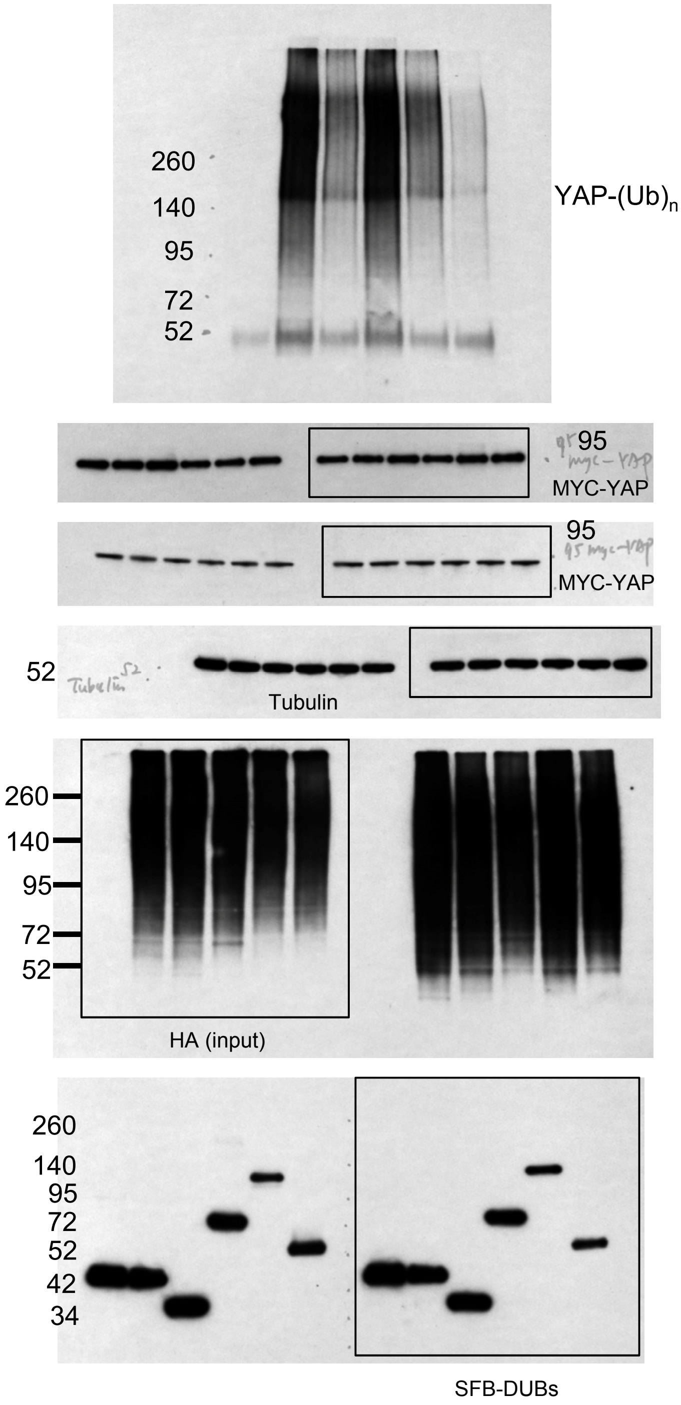

Supplementary Figure 4e

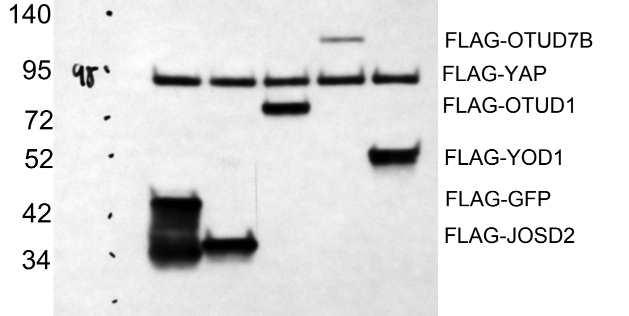

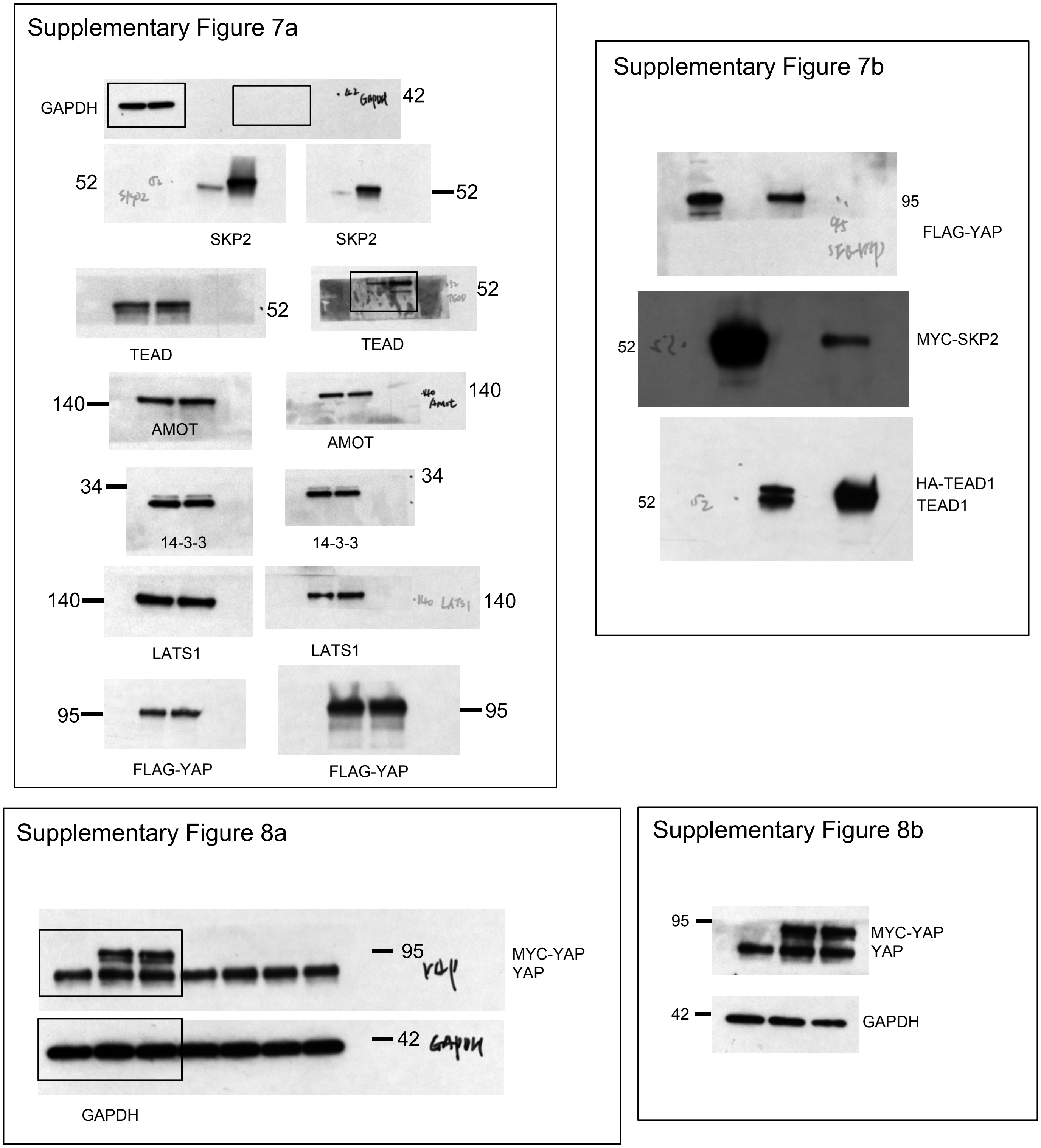

Supplementary Figure 9. Uncropped blots and gels.

Supplementary Table 1. Primers for qPCR

Gene	Primer sequence (5′-3′)
CTGF-F	CCAATGACAACGCCTCCTG
CTGF-R	GAGCTTTCTGGCTGCACCA
CYR61-F	AGCCTCGCATCCTATACAACC
CYR61-R	GAGTGCCGCCTTGTGAAAGAA
ANKRD1-F	CACTTCTAGCCCACCCTGTGA
ANKRD1-R	CCACAGGTTCCGTAATGATTT
ACTB-F	GATCATTGCTCCTCCTGAGC
ACTB-R	ACTCCTGCTTGCTGATCCAC
SKP2-F	GACTTAAGTGATAGTGTCATGC
SKP2-R	ATAGGTGTTGGAGGTAGTTG
OTUD1-F	AGCTTTGGGTGATTTTACTG
OTUD1-R	GCTGTTTGTCAAAATGGAAC
